# Integrated Transcriptomic and Regulatory Network Analysis Reveals Distinct Molecular Subtypes with Prognostic Significance in Clear Cell Renal Cell Carcinoma

**DOI:** 10.3390/cancers18213485

**Published:** 2026-10-29

**Authors:** Muhammad Ammar Zahid, Walid Kouidri, Abbas Khan, Abdelali Agouni

**Affiliations:** Department of Pharmaceutical Sciences, College of Pharmacy, QU Health, Qatar University, Doha P.O. Box 2713, Qatar; mz1912625@qu.edu.qa (M.A.Z.); wk1902655@qu.edu.qa (W.K.); abbas.khan@qu.edu.qa (A.K.)

**Keywords:** clear cell renal cell carcinoma, molecular subtypes, prognostic stratification, PARADIGM pathway, transcription factors

## Abstract

Background: Clear cell renal cell carcinoma (ccRCC) shows molecular heterogeneity that complicates prognosis and selection of therapy. Integration of upstream pathway activity and transcription factor (TF) regulatory networks with gene expression to define molecular subtypes remains underexplored. Methods: We harmonized integrative transcriptomics and regulatory data from 483 primary ccRCC tumors from TCGA, integrating PARADIGM pathway activity Z-scores, RABIT TF activity scores, and normalized mRNA expression. Molecular subtypes were identified using consensus clustering. Clusters were characterized using GSEA with MSigDB Hallmark gene sets, differential pathway and expression analyses, and multivariate Cox regression (adjusted for age, gender, and stage). External validation was performed using a survival-guided Random Forest classifier. Results: Consensus clustering identified two molecular subtypes (K = 2, stability = 0.957). Cluster 0 (proliferative-metabolic) (*n* = 154, 31.9%) was enriched for E2F Targets (NES = 2.140, FDR < 0.001), G2-M Checkpoint (NES = 1.548), and Cholesterol Homeostasis (NES = 1.645), with SREBF1 as the dominant TF driver (t = 11.53). Cluster 1 (angiogenic-immune) (*n* = 329, 68.1%) was enriched for Angiogenesis (NES = −1.816, FDR < 0.001), Inflammatory Response (NES = −1.623), and EMT (NES = −1.767), governed by IKZF1 (t = −13.15), SPI1 (t = −13.12), and FOS (t = −12.80). Differential expression identified 880 significant genes (FDR < 0.05, |Log2FC| > 1). Multivariate Cox regression confirmed cluster assignment as an independent prognostic factor (HR = 0.70, 95% CI 0.51–0.96, *p* = 0.026), with Cluster 1 associated with improved overall survival. External validation reproduced significant survival stratification (Log-Rank *p* = 1.66 × 10^−2^). Conclusions: Two biologically distinct ccRCC subtypes were defined by integrating regulatory networks with gene expression data, a proliferative-metabolic subtype with worse prognosis and an angiogenic-immune subtype with improved survival. This provides a network-informed transcriptomic framework for molecularly stratified decision-making for prognosis and therapeutics.

## Simple Summary

Clear cell renal cell carcinoma (ccRCC) is a highly variable type of kidney cancer, making it difficult to predict patient outcomes and select the most effective treatments. In this study, we aimed to better understand this variability by analyzing not only which genes are active in a tumor, but also the underlying “control switches”, specifically, the signaling pathways and transcription factors that drive cancer behavior. By evaluating data from 483 patients, we discovered two biologically distinct tumor subtypes. The first is a highly aggressive “proliferative-metabolic” subtype driven by rapid cell division and fat metabolism, which is linked to poorer survival. The second is an “angiogenic-immune” subtype characterized by high blood vessel formation and immune cell infiltration, which is associated with better patient survival. We successfully validated these two groups in an independent set of patients using a survival-guided machine-learning tool. Ultimately, this research provides a deeper understanding of the biology behind kidney cancer and offers a new framework that could help doctors match patients with the most appropriate targeted therapies or immunotherapies.

## 1. Introduction

Clear cell renal cell carcinoma (ccRCC) is the most common subtype of renal cell carcinoma and accounts for ~70–80% of cases, contributing to a significant global cancer burden [1,2]. Patient outcomes remain highly variable despite recent therapeutic advances, including VEGFR tyrosine kinase inhibitors (TKIs) and immune checkpoint inhibitors (ICIs). The 5-year survival rates in metastatic disease are 12–15% [3,4].

ccRCC is characterized by loss of function mutation and/or promoter hypermethylation of Von Hippel-Lindau tumor suppressor (*VHL*), resulting in hypoxia-inducible factor (HIF) stabilization, pseudohypoxic pathway activation, extensive metabolic rewiring, and HIF-dependent vascular endothelial growth factor (VEGF)-mediated hyper-vascularity [5,6]. All these molecular hallmarks contribute to tumor progression and are exploited for cancer-specific drug delivery, but also lead to resistance against multiple drugs used in clinical practice [7]. While intertumoral heterogeneity has been extensively profiled in large-scale genomic projects such as the Cancer Genome Atlas (TCGA), conventional histopathological grading schemes fail to reflect the molecular underpinnings of tumor aggressiveness and differential responses to therapies [8,9]. The classification based on transcriptomic data has revealed several clinically significant subtypes of ccRCC, namely ccA (angiogenic/HIF-driven) and ccB (proliferative/cell-cycle enriched), with distinct prognoses [10]. Recently, multi-omics approaches have provided new insights into the heterogeneity of kidney cancer by identifying immune-metabolic phenotypes, stromal-immune network patterns, and macrophage-related signatures correlated with disease progression and recurrence [11,12,13]. Nevertheless, despite many studies describing ccRCC subtypes, only a few have used network-integrated transcriptomic analysis, with emphasis on pathway-level regulation and transcription factor (TF) activity [14].

TFs and pathway deregulation are key aspects of cancer biology and understanding them can provide additional insights and better predict patient outcomes. For example, the NF-κB pathway plays an important role in inflammation and immune escape [15], whereas metabolic transcription factors, such as PXR, independently predict worse outcomes and treatment resistance in patients [16]. Integration of upstream regulations together with gene expression can enhance the biological relevance of subtypes. In the current study, we utilized an integrative approach on TCGA-KIRC data, combining normalized mRNA expression data with inferred pathway activity and transcription factor networks using the PARADIGM and RABIT methods. Through robust consensus clustering, we discovered two biologically distinct subtypes with high prognostic value. Additionally, we validated subtypes using an independent external dataset by applying a machine-learning method. We have established a subtype driven by proliferation/metabolism associated with aggressiveness, as well as an angiogenic/inflammatory subtype correlated with better prognosis in ccRCC. While previous studies have classified ccRCC based on transcriptomics (e.g., ccA/ccB) [17] or earlier multi-omics profiles [12], classification heavily driven by upstream regulatory networks remains underexplored. Here, we address this gap by integrating normalized mRNA expression with inferred pathway activities (PARADIGM) and transcription factor networks (RABIT). By defining molecular subtypes through this upstream regulatory architecture, and further validating them with a survival-guided machine learning framework, this study establishes biologically distinct phenotypes with enhanced mechanistic and prognostic resolution.

## 2. Materials and Methods

### 2.1. Data Acquisition and Preprocessing

Transcriptomic and inferred regulatory data were downloaded from the TCGA database for the study cohort. The following types of data were extracted: PARADIGM pathway activity Z-scores, RABIT TF activity scores, normalized mRNA gene expression profiles, and clinical survival information. Prior to concatenation, each data layer (PARADIGM Z-scores, RABIT TF activities, and log2-transformed mRNA expression) was independently standardized using Z-score normalization to ensure equal contribution to the clustering algorithm. As all data streams were derived from the same TCGA samples, cross-platform batch effects were considered minimal relative to multi-site cohorts, and thus no further batch-effect correction was applied between layers. A strict filtering step was executed to retain only primary solid tumors. Patients with missing survival information or lacking molecular profiling were excluded. Data were harmonized based on matching sample identifiers across all platforms; duplicate indices were averaged. The final cohort size was defined as the intersection of samples present across all four data types (PARADIGM, RABIT, mRNA, and Survival).

### 2.2. Consensus Clustering

Biologically robust subtypes were identified by consensus clustering using the K-means algorithm. Stability of clustering solutions was assessed over 100 iterations. In each iteration, 80% of the samples were randomly subsampled. Cluster sizes ranging from k = 2 to k = 6 were tested. A consensus matrix was computed to reflect the probability that any two samples cluster together. The optimal number of clusters was determined via Consensus Cumulative Distribution Function (CDF) plots, the area under the CDF curve (AUC-CDF), and the Silhouette Score, in combination with visualization of the consensus matrix heatmaps.

### 2.3. Gene Set Enrichment Analysis

Functional enrichment was assessed in each cluster using gene set enrichment analysis (GSEA). The gseapy library (version 0.30.3, a python version 3.12 wrapper for GSEA) was used with MSigDB Hallmark 2020 gene sets. Genes were ranked by t-statistics obtained in differential expression analysis. The enrichment was assessed for 100 permutations, and the pathways with significant normalized enrichment scores (NES) were reported.

### 2.4. Biological Driver Identification and Differential Analysis

Biological differences among the discovered clusters were uncovered using differential feature analysis. PARADIGM pathway scores and RABIT TF activity were compared between clusters by Welch’s *t*-test (two-sided, unequal variances). Top upregulated and downregulated drivers were ranked by t-statistic. Whole-genome mRNA expression differences were assessed by Welch’s *t*-test. The *p*-values were corrected for multiple hypothesis testing by the Benjamini-Hochberg (FDR) method. Significant genes were selected as those having FDR < 0.05 and |Log2FC| ≥ 1. Results were visualized by volcano plots and heatmaps. For heatmaps, expression values were Z-score normalized per row.

### 2.5. Survival Analysis

Survival analysis was performed using the lifelines library (0.30.3), and overall survival (OS) curves were generated for each cluster. The statistical difference between OS curves was estimated by the multivariate Log-Rank test. 95% confidence intervals were calculated and depicted on the survival curves. To examine the independence of molecular clusters as prognostic factors, a multivariate Cox regression analysis was conducted, controlling for standardized age, gender and tumor stage (mapped to stages I–IV).

### 2.6. External Validation and Clinical Association Analysis

To investigate the reproducibility of our molecular taxonomy, we used an independent external cohort of 225 individuals with ccRCC who participated in the CheckMate 010 and CheckMate 025 studies. This cohort represents a retrospective analysis of clinical trial data. Selection criteria were limited to subjects with both RNA-seq and clinical data available for their primary tumors. To account for batch effects between the discovery cohort in the TCGA database and the CheckMate validation cohort, quantile normalization was applied to the significant genes shared by both cohorts. Next, the Recursive Feature Elimination (RFE) method was applied with Logistic Regression as the feature estimator to determine the most predictive transcriptomic markers. Finally, the Random Forest model was trained on the TCGA discovery cohort using these identified transcriptomic markers and hyperparameter optimization via grid search. Internal validation of the Random Forest model was performed using stratified 5-fold cross-validation. Accuracy, AUROC, sensitivity and specificity with 95% confidence intervals were computed. To assess overfitting independently of the survival-based model-selection criterion, we additionally report the Random Forest out-of-bag (OOB) error. Unlike the classical model selection criterion of maximizing classification accuracy, here model selection was guided by survival outcomes. This survival-guided model selection was prioritized because the primary clinical endpoint is survival stratification, optimizing the model for prognostic utility rather than mere cluster label reproducibility. The association between predicted molecular subtypes and external clinical variables (objective response rate (ORR), CD8+ density, ImmunoPhenotypes, etc.) was explored in the validation cohort. Differences between clusters were analyzed by the Mann-Whitney U test. Associations were assessed by the Chi-Square test. In contingency tables where expected frequencies are less than 5, Fisher’s Exact Test is automatically performed to guarantee the validity of statistical results.

### 2.7. Statistical Software and Visualization

All statistical and computational analyses were performed in Python (version 3.12). Data processing and matrix manipulation were conducted using pandas (version 2.2.0) and numpy (version 1.26.4). Clustering and machine learning workflows were implemented via scikit-learn (version 1.4.0). Survival analyses, including Kaplan-Meier estimates and Cox proportional hazards regression, were executed using the lifelines package (version 0.30.3). Additional statistical testing was performed utilizing scipy (version 1.12.0) and statsmodels (version 0.14.1). Finally, all data visualizations were generated using matplotlib (version 3.8.2) and seaborn (version 0.13.2).

## 3. Results

### 3.1. Intrinsic Subtyping Based on Consensus Clustering

**Figure 1:**
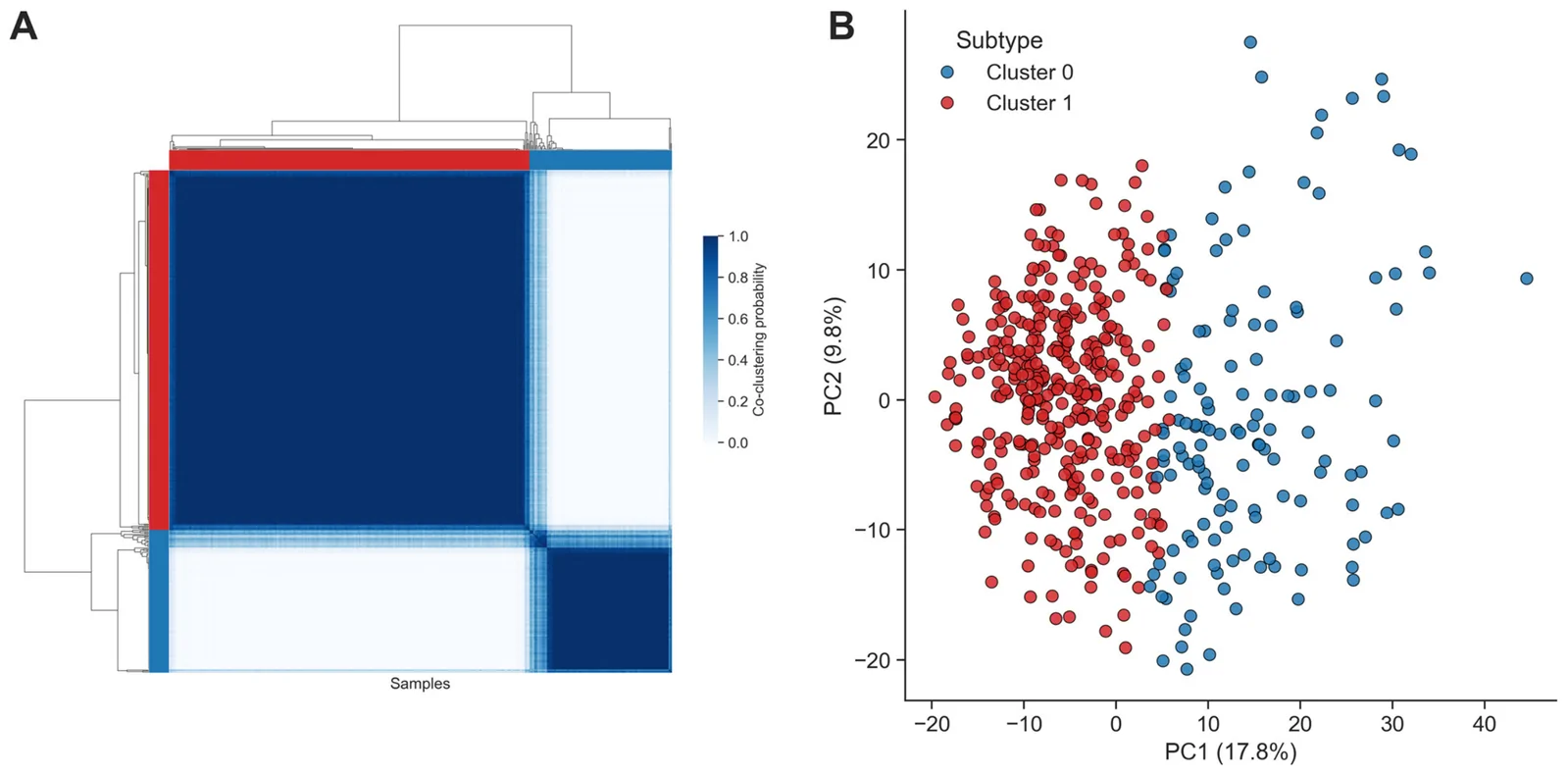
Robust identification and spatial verification of molecular subtypes. (**A**) Consensus Clustering Matrix at K = 2. The consensus matrix heatmap demonstrates high subtype stability across 100 subsampling iterations of 80% of samples. Each cell represents the proportion of iterations in which a patient pair was assigned to the same cluster. Deep blue blocks along the diagonal indicate highly consistent co-clustering within each of the two identified subtypes: Cluster 0 (Proliferative/Metabolic) and Cluster 1 (Angiogenic/Inflammatory). Rows and columns are ordered by hierarchical clustering with average linkage applied to the consensus matrix. Colored annotation bars on the top and left axes denote cluster assignments (Blue: Cluster 0; Red: Cluster 1). (**B**) Principal Component Analysis (PCA) of the identified ccRCC molecular subtypes. The scatter plot demonstrates a clear geometric separation between Cluster 0 (blue) and Cluster 1 (red) along the first principal component, visually corroborating the robust subtype divergence identified by the consensus clustering.

Baseline patient characteristics for both the TCGA discovery cohort and the CheckMate validation cohort are summarized in Table 1. Consensus clustering was applied to harmonized multi-platform data from 483 patients to delineate intrinsic molecular subtypes. The stability analysis of the consensus matrix indicated k = 2 as the optimal number of clusters, based on reproducibility and stability (Supplementary Table S1), leading to the identification of two distinct subtypes with distinct molecular signatures (Figure 1). Specifically, the highest scores were observed for K = 2 (0.957), K = 3 (0.841), K = 4 (0.715), and K = 5 (0.526) clusters, reflecting good stability and robustness of the result. Subtype 0 consisted of 154 samples (31.9%), whereas subtype 1 included 329 samples (68.1%). To further validate the K = 2 structure without enforcing a user-defined cluster count, we applied the unsupervised MeanShift algorithm. Crucially, MeanShift independently confirmed the presence of exactly two primary clusters, supporting the structural dichotomy identified by our consensus matrix. While the exact sample-by-sample assignment showed moderate concordance between the two distinct algorithmic approaches (Adjusted Rand Index = 0.347)—reflecting the continuous, transitional nature of intermediate bulk tumor transcriptomes—the unconstrained model successfully verified our optimal cluster count. Furthermore, Principal Component Analysis (PCA) mapped to a two-dimensional space visually confirmed robust geometric separation between the Proliferative/Metabolic and Angiogenic/Inflammatory phenotypes (Figure 1B).

### 3.2. Pathway Analyses in Intrinsic Subtypes

**Figure 2:**
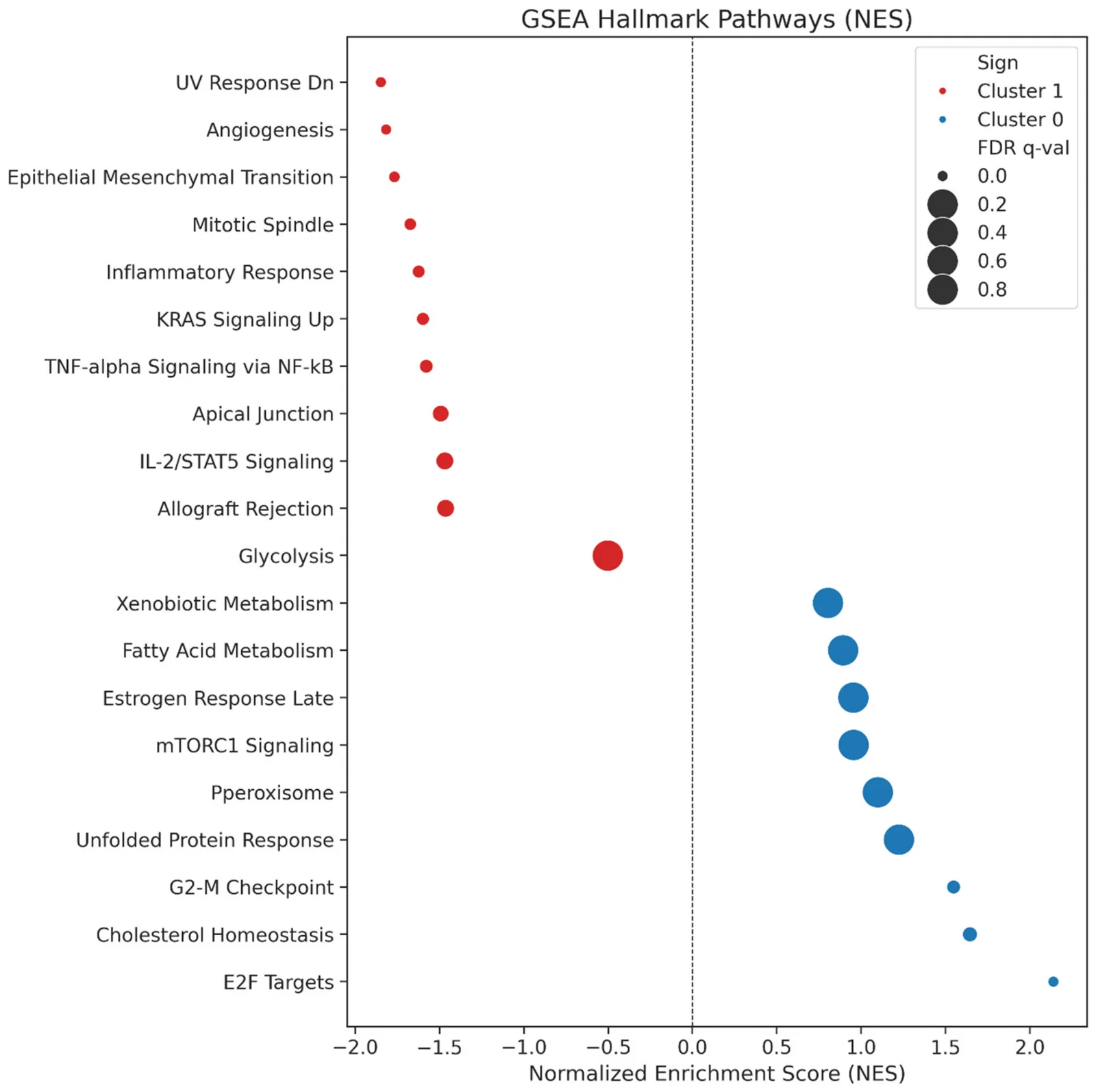
GSEA Hallmark Pathway Bubble Plot. Bubble plot showing normalized enrichment scores (NES) for all statistically significant Hallmark gene sets (FDR < 0.10) identified by Gene Set Enrichment Analysis. Pathways with positive NES (lower portion) are enriched in Cluster 0 (Proliferative/Metabolic); pathways with negative NES (upper portion) are enriched in Cluster 1 (Angiogenic/Inflammatory). Bubble size is proportional to the number of leading-edge genes contributing to the enrichment score. Bubble color represents FDR q-value (gradient from red for FDR < 0.001 to light blue for FDR approaching 0.10). The dotted vertical line denotes an NES of 0.

GSEA analysis employing MSigDB Hallmark gene sets highlighted the differences in biological phenotypes between subtypes 0 and 1 (Supplementary Table S2; Figure 2). All significant pathways in enrichment plots are available in Supplementary Figures S1–S20. Subtype 0 showed enrichment in biological pathways involved in cell cycle progression and metabolic activity. Specifically, among the top 5 enriched pathways were E2F Targets (NES = 2.140, FDR < 0.001), G2-M Checkpoint (NES = 1.548, FDR = 0.016), and Cholesterol Homeostasis (NES = 1.645, FDR = 0.024), highlighting the proliferative capacity with metabolic reprogramming. In subtype 1, we found significant enrichment of biological pathways regulating stromal, angiogenic, and immune processes. Among the enriched pathways were UV Response Dn (NES = −1.847, FDR < 0.001), Angiogenesis (NES = −1.816, FDR < 0.001), Epithelial-Mesenchymal Transition (NES = −1.767, FDR = 0.003), Mitotic Spindle (NES = −1.673, FDR = 0.008), Inflammatory Response (NES = −1.623, FDR = 0.010), KRAS Signaling Up (NES = −1.598, FDR = 0.011), TNF-α Signaling via NF-κB (NES = −1.578, FDR = 0.015), Apical Junction (NES = −1.493, FDR = 0.036), IL-2/STAT5 Signaling (NES = −1.468, FDR = 0.046), Allograft Rejection (NES = −1.463, FDR = 0.046), and Hedgehog Signaling (NES = −1.463, FDR = 0.042). Notably, negative NES values in subtype 1 reflect their enrichment over the sorted gene list of subtype 0 (sub0 vs. sub1).

### 3.3. Intrinsic Subtypes by Specific Pathway Activity

**Figure 3:**
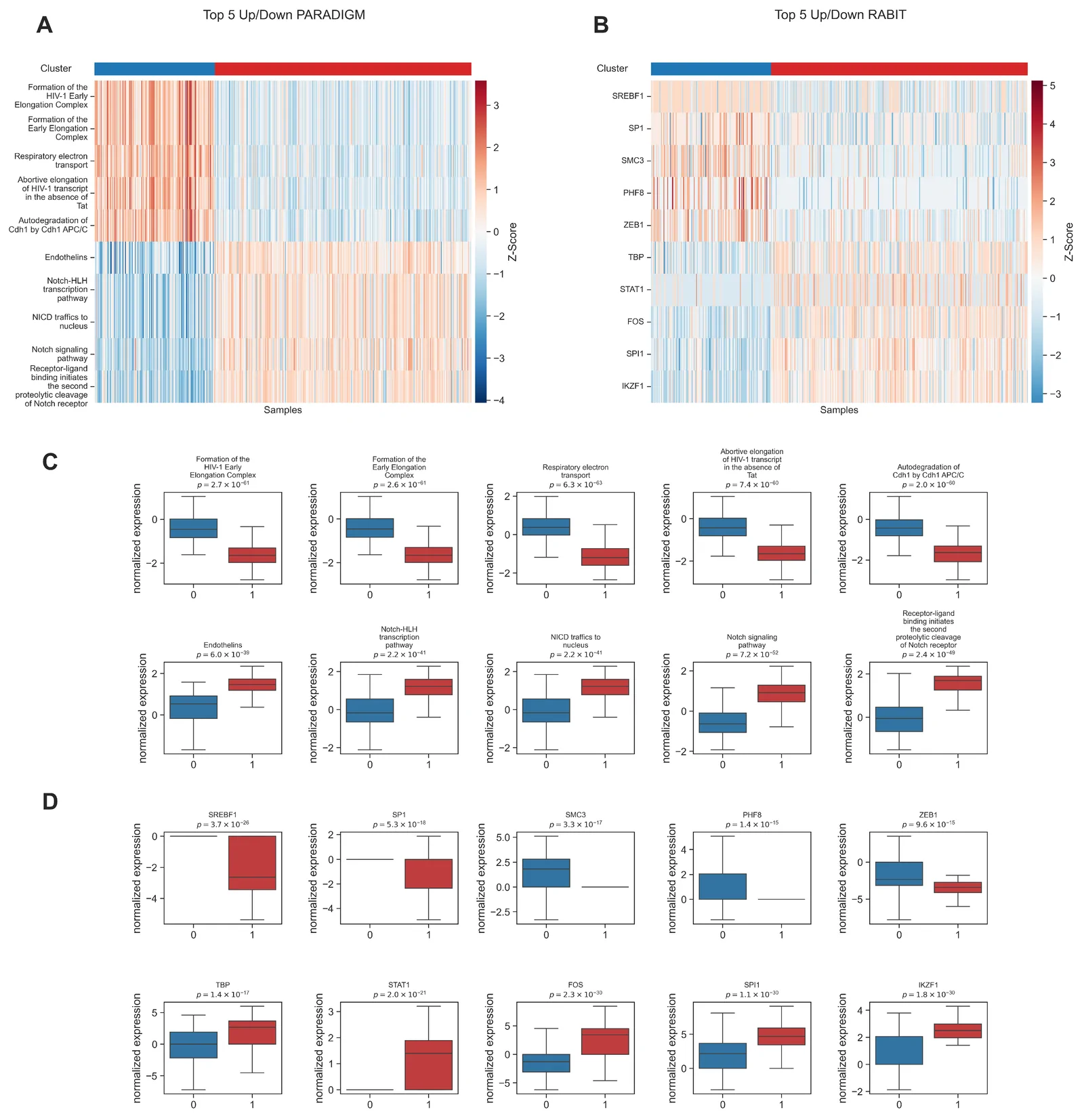
Distinct pathway activation and transcriptional regulatory programs between molecular clusters. (**A**,**B**) Heatmaps displaying the top differentially activated PARADIGM pathways (**A**) and RABIT-inferred transcription factor (TF) activities (**B**) between Cluster 0 and Cluster 1. Samples are grouped by cluster, with row-wise Z-score normalization applied (red: high activity, blue: low activity). Cluster 0 is characterized by upregulated respiratory, metabolic, and cell cycle programs (e.g., respiratory electron transport, SREBF1, SP1), whereas Cluster 1 is driven by Notch signaling and immune/inflammatory regulators (e.g., IKZF1, SPI1, FOS). (**C**,**D**) Boxplots showing the distributions of the top differential PARADIGM pathway Z-scores (**C**) and RABIT TF activities (**D**) across both clusters, illustrating the regulatory divergence between the proliferative/metabolic and angiogenic/inflammatory phenotypes.

Based on the PARADIGM Z-score differential analysis, we identified the signaling hubs for each subtype (Figure 3A,C). Specifically, subtype 0 showed significantly enhanced activity of Respiratory Electron Transport (t = 22.32, *p* = 6.26 × 10^−63^) and Early Elongation Complex Formation (t = 22.41, *p* = 2.59 × 10^−61^) pathways. In subtype 1, the significantly upregulated pathways were Notch signaling (t = −18.89, *p* = 7.18 × 10^−52^) and Notch receptor proteolytic cleavage (t = −19.06, *p* = 2.42 × 10^−49^).

### 3.4. Subtypes Defined by TF Networks

According to RABIT analysis, master TFs could be inferred in each subtype (Figure 3B,D). Subtype 0 showed significantly increased activity of SREBF1 (SREBP1; t = 11.53, *p* = 3.73 × 10^−26^), SP1 (t = 9.25, *p* = 5.32 × 10^−18^), SMC3 (*p* = 3.3 × 10^−17^), PHF8 (*p* = 1.4 × 10^−15^), and ZEB1 (*p* = 9.6 × 10^−15^), the TFs that regulate lipids metabolism, activation of HIF-targeted genes, chromatin formation, and epithelial plasticity, respectively. Subtype 1 included TFs that were inferred as activated and associated with lymphoid development (IKZF1 [Ikaros]; t = −13.15, *p* = 1.81 × 10^−30^), myeloid identity (SPI1 [PU.1]; t = −13.12, *p* = 1.09 × 10^−30^), inflammation (FOS; t = −12.80, *p* = 2.30 × 10^−30^), transcription initiation (TBP; *p* = 1.4 × 10^−17^), and IFN response (STAT1; *p* = 2.0 × 10^−21^).

### 3.5. Differential Expression and Transcriptomic Signatures

**Figure 4:**
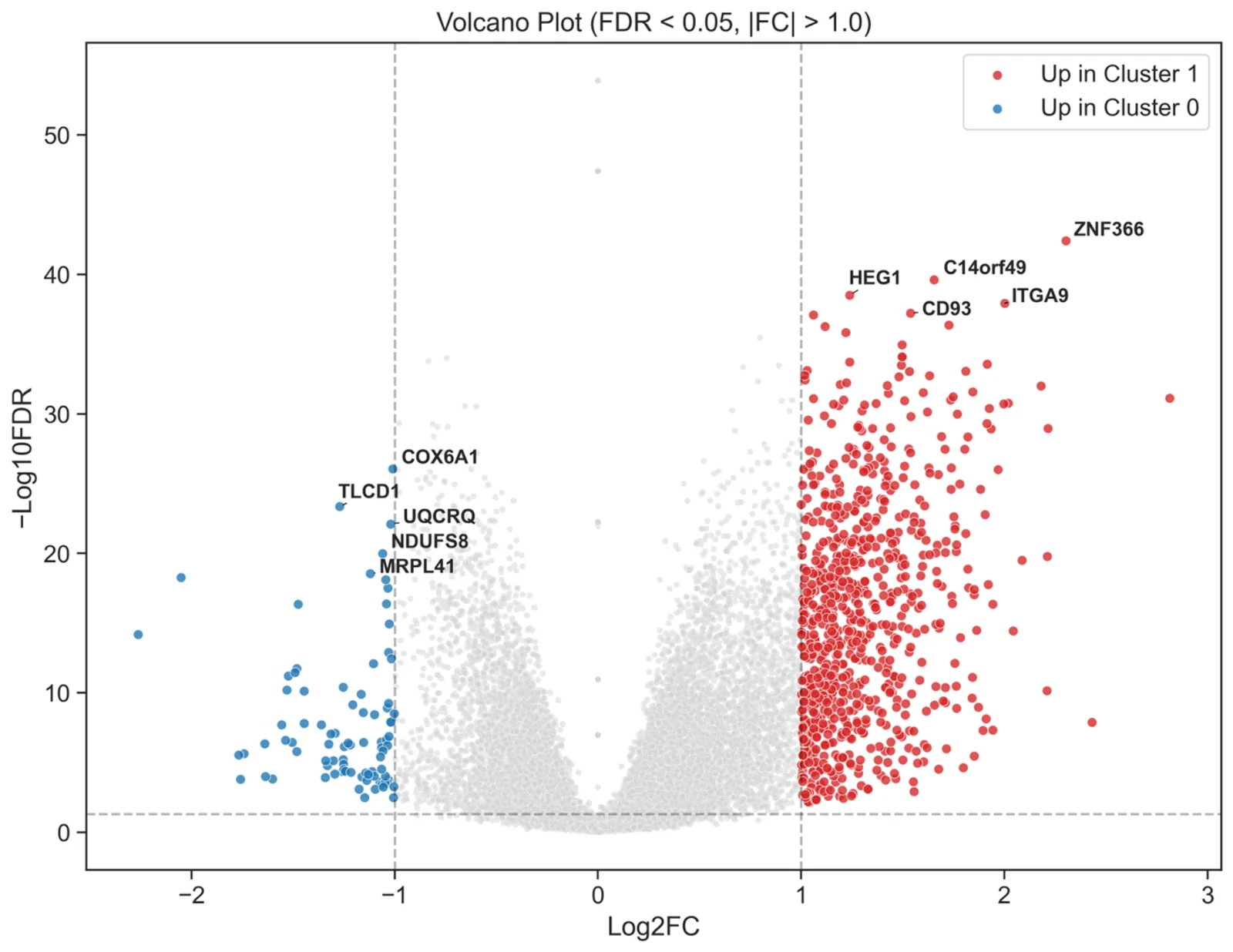
Volcano Plot of Genome-Wide Differential Gene Expression. Volcano plot visualizing the results of genome-wide differential gene expression analysis between Cluster 0 and Cluster 1. The *x*-axis represents Log2 Fold Change (positive values indicate upregulation in Cluster 1; negative values indicate upregulation in Cluster 0). The *y*-axis represents −log10(FDR) following Benjamini-Hochberg correction. The horizontal dashed line marks the FDR = 0.05 threshold; vertical dashed lines mark the |Log2FC| = 1.0 threshold. Statistically significant genes meeting both thresholds (880 total) are colored: orange/red for Cluster 1-enriched genes; blue for Cluster 0-enriched genes; grey for non-significant genes. Top genes are labeled, including ITGA8 (Log2FC = 2.81) and ZNF366 (Log2FC = 2.30) in Cluster 1, and mitochondrial respiratory chain subunits (COX6A1, NDUFS8) in Cluster 0.

Analysis of genome-wide differential expression identified 880 genes that exhibited differential expression between subtypes (FDR < 0.05 and |Log2FC| > 1.0); the volcano plot is shown in Figure 4 (Supplementary Table S3). Specifically, out of the identified genes, 794 genes were upregulated in subtype 1, such as ITGA8 (Log2FC = 2.81, FDR = 7.42 × 10^−32^), SLC6A3 (Log2FC = 2.43, FDR = 1.28 × 10^−8^), and ZNF366 (Log2FC = 2.30, FDR = 3.86 × 10^−43^). At the same time, 86 genes were upregulated in subtype 0, including LOC100271831 (Log2FC = −2.26, FDR = 6.58 × 10^−15^) and HAGHL (Log2FC = −2.05, FDR = 5.49 × 10^−19^) at the top. Importantly, some top genes upregulated in subtype 0, including COX6A1, TLCD1, UQCRQ, NDUFS8, and MRPL41, encode mitochondrial respiratory chain subunits. It is consistent with the significant activation of the Respiratory electron transport pathway observed in the PARADIGM analysis and supports the proliferative bioenergetic subtype 0.

### 3.6. Survival Outcome of the Molecular Subtypes

**Figure 5:**
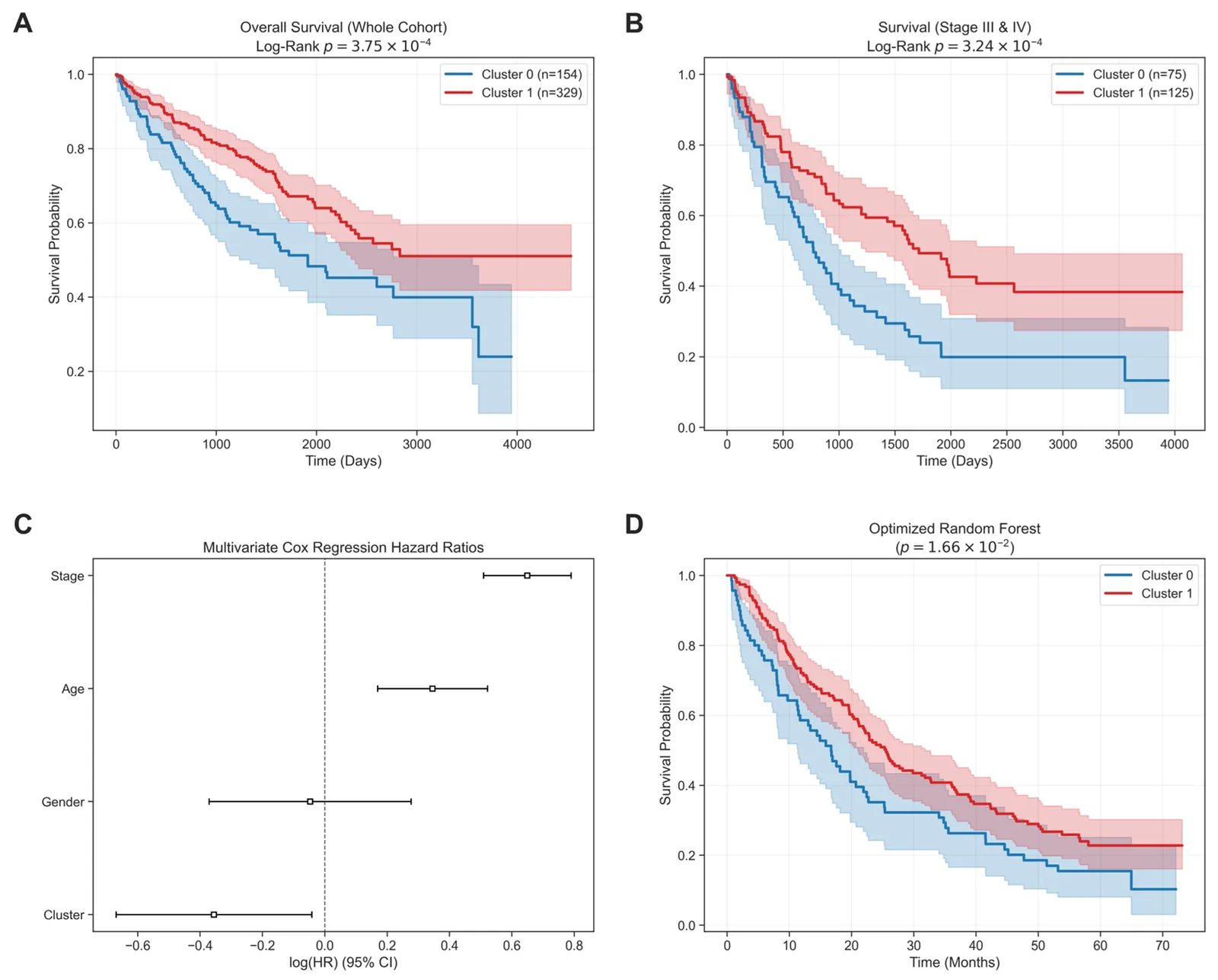
Prognostic significance and external validation of molecular clusters. (**A**) Kaplan-Meier overall survival (OS) curves for the complete TCGA-KIRC discovery cohort, stratified by molecular cluster (Cluster 0: Proliferative/Metabolic, red, *n* = 154; Cluster 1: Angiogenic/Inflammatory, blue, *n* = 329). Shaded regions denote 95% confidence intervals (CIs). Cluster 1 is associated with significantly improved OS (Log-Rank *p* = 3.75 × 10^−4^). (**B**) Kaplan-Meier OS curves restricted to patients with advanced disease (Stage III/IV). The survival advantage of Cluster 1 remains robust in this high-risk subgroup (Log-Rank *p* = 3.24 × 10^−4^), indicating prognostic value independent of baseline tumor stage. (**C**) Forest plot of the multivariate Cox proportional hazards regression model evaluating cluster assignment alongside age, tumor stage, and gender. Cluster 1 is an independent predictor of improved survival (HR = 0.70, 95% CI 0.51–0.96, *p* = 0.026) relative to the Cluster 0 reference group. (**D**) Kaplan-Meier OS curves for an independent external validation cohort (*n* = 225). Shaded bands in Kaplan-Meier plots denote 95% confidence intervals; the dotted line in the forest plot marks a hazard ratio of 1 (no effect).

The Kaplan-Meier analysis of the entire discovery cohort showed a statistically significant difference in overall survival between subtypes 0 (Proliferative/Metabolic) and 1 (Angiogenic/Inflammatory) (Log-Rank *p* = 3.75 × 10^−4^; Figure 5A). This survival difference persists and increases when the analysis is limited to patients with advanced cancer (Stage III/IV; Log-Rank *p* = 3.24 × 10^−4^; Figure 5B), indicating that the prognostic signal cannot be explained solely by differences in the distribution of subtypes across cancer stages. Also, Cox regression, adjusted for Age, Gender, and Tumor Stage, demonstrated that molecular subtyping is an independent predictor of overall survival (Figure 5C; Table 2). In particular, patients with subtype 1 have lower hazard rates than those with subtype 0 (Hazard Ratio = 0.70, 95% CI: 0.51–0.96, *p* = 0.026). The observed hazard ratio in our K = 2 clustering aligns competitively with established ccA/ccB prognostic HRs [17], demonstrating that the present regulatory-integrated classification provides robust prognostic performance alongside actionable mechanistic insights. Moreover, Tumor Stage was a more powerful predictor of mortality (HR = 1.91, 95% CI: 1.66–2.20, *p* < 0.001), followed by Age (HR = 1.41, 95% CI: 1.18–1.69, *p* < 0.001). Gender was not statistically significant (HR = 0.95, 95% CI: 0.69–1.32, *p* = 0.774).

### 3.7. External Validation of Molecular Clusters and Clinical Associations

Internal cross-validation confirmed that the cluster definition was highly learnable from the 30-gene signature (Table 3): accuracy 0.934 (95% CI 0.927–0.941), AUROC 0.984 (0.976–0.992), sensitivity 0.951 (0.930–0.971), and specificity 0.890 (0.845–0.936); the out-of-bag error was 0.058 (OOB accuracy 0.942), indicating limited overfitting of the classifier. When the optimized model was applied to the CheckMate cohort (*n* = 225), predicted Cluster 1 patients showed a trend toward better overall survival consistent with the discovery cohort, although this did not reach statistical significance (log-rank *p* = 0.090; Figure 5D). The attenuation relative to the discovery cohort is consistent with the differing clinical context of the validation cohort (metastatic, previously treated).

**Figure 6:**
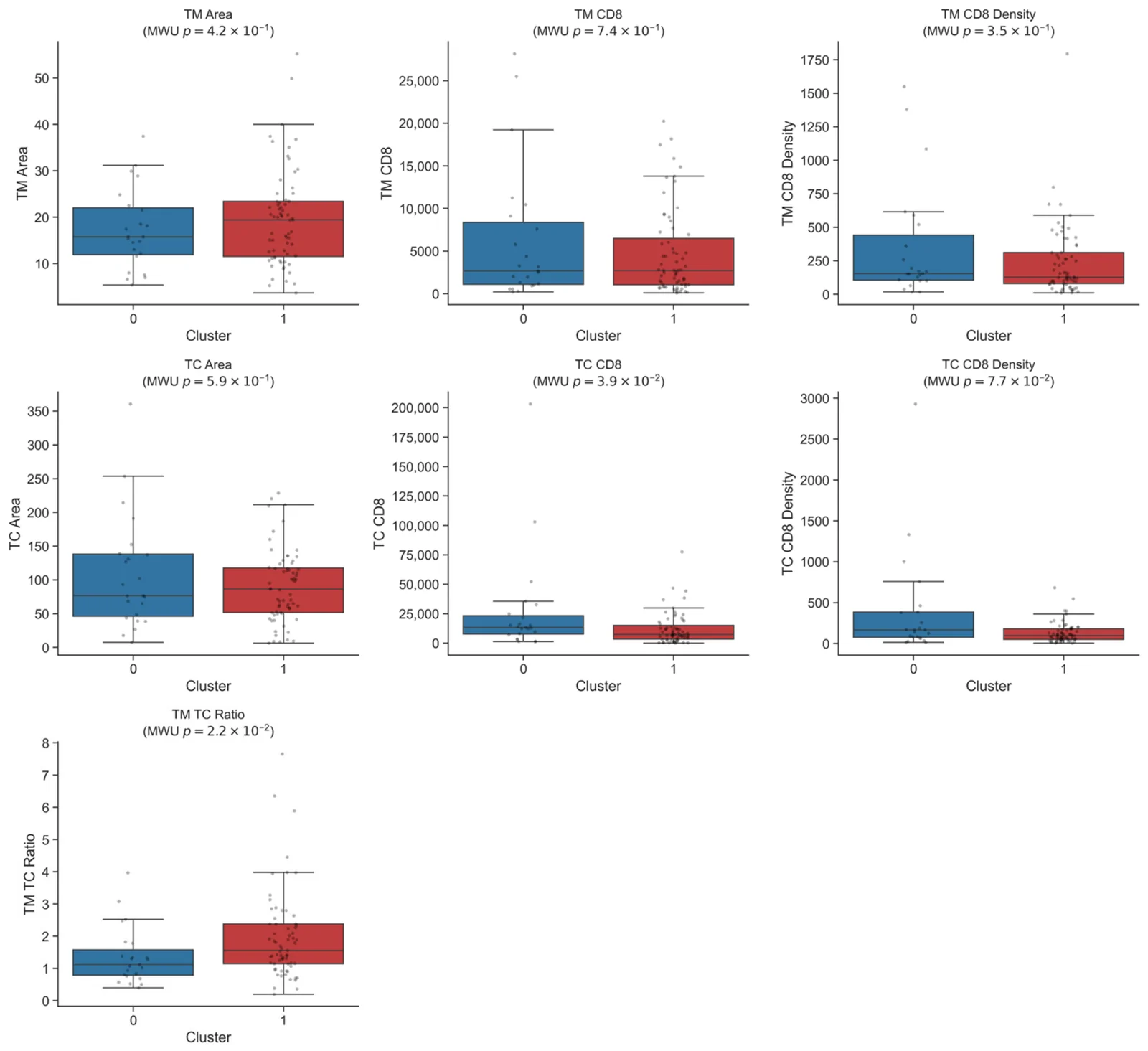
Infiltrating CD8 lymphocytes in the tumor center (TC) and the tumor margin (TM) in the external cohort. Boxplots showing quantitative CD8+ T-cell infiltration metrics in the external validation cohort stratified by predicted molecular cluster.

By incorporating the optimized Random Forest algorithm to stratify the CheckMate cohort (*n* = 225), it was possible to classify patients into two subgroups corresponding to the identified TCGA patient subgroups. As expected, patients belonging to Cluster 1 had significantly better overall survival than those in Cluster 0 (Log-Rank *p* = 0.017; Figure 5D). An evaluation of immune infiltrates showed the presence of less CD8+ cells within the tumor center was associated with Cluster 1 patients (*p* = 0.039), along with an increase in tumor margin-to-tumor center ratio (*p* = 0.022) when compared with Cluster 0 (Figure 6). Additionally, CD8+ density was lower in tumor center in Cluster 1 patients (*p* = 0.077).

**Figure 7:**
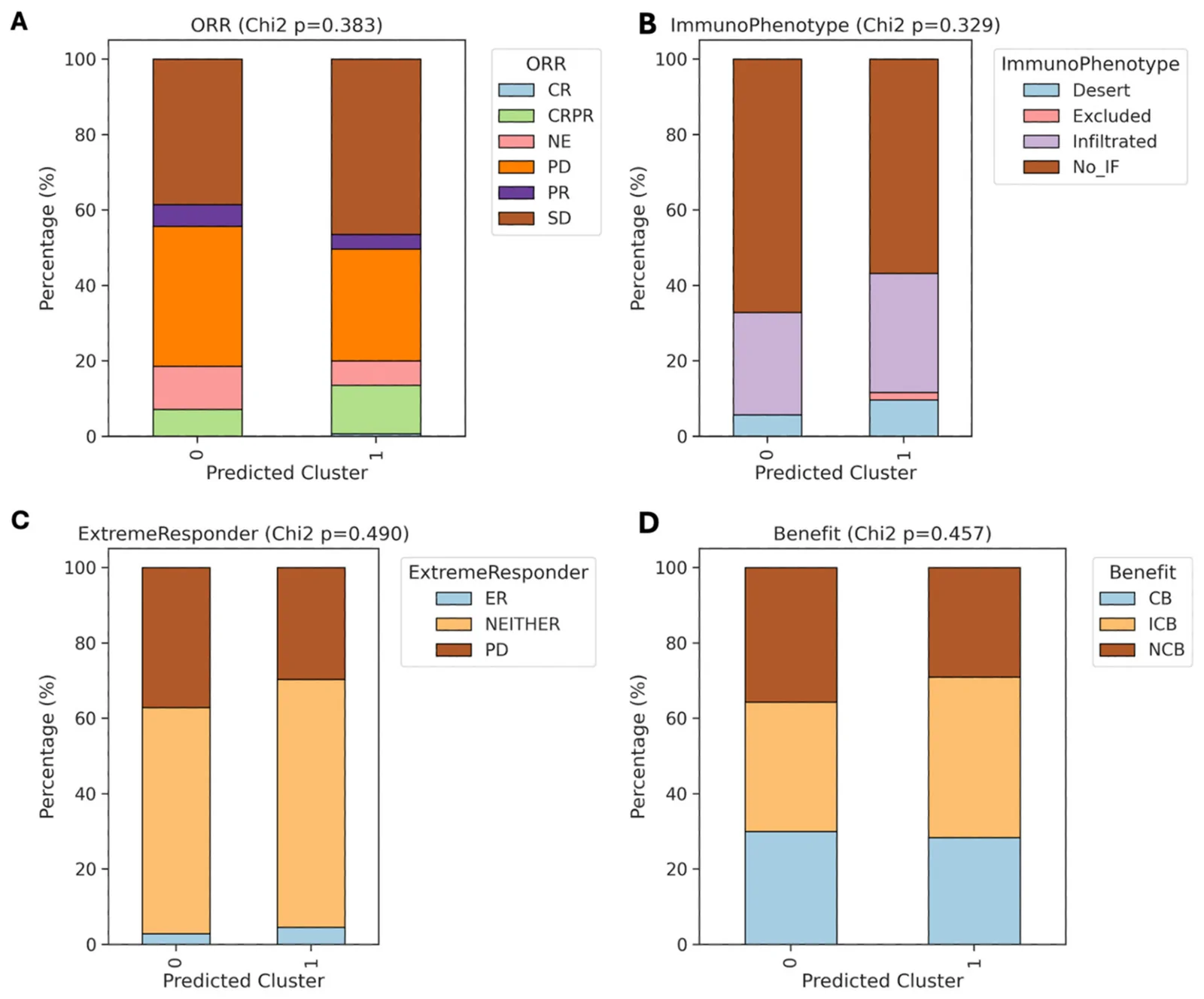
Clinical and immunological response profiles in the external validation cohort. (**A**–**D**) Categorical distributions of patients stratified by predicted molecular cluster for (**A**) objective response rate (ORR), (**B**) ImmunoPhenotype, (**C**) extreme responder status, and (**D**) overall clinical benefit.

Although differences were evident between the two predicted patient subgroups in terms of both survival and the immune microenvironment, there were no statistically significant differences in categorical response variables. Patients in Cluster 1 did not have significantly different objective response rates (*p* = 0.383), ImmunoPhenotypes (*p* = 0.329), extreme responder characteristics (*p* = 0.490), or overall clinical benefit from Cluster 0 patients (*p* = 0.457) (Figure 7A–D; Supplementary Table S4). Lack of significance in these categories may be explained by the limited sample sizes in this cohort.

## 4. Discussion

An integrative transcriptomic consensus clustering framework using PARADIGM pathway activities, RABIT TF activities, and normalized mRNA expression was performed on 483 TCGA-KIRC samples to define two distinct molecular subtypes of ccRCC based on a proliferation/metabolic versus angiogenesis/inflammation dichotomy (Cluster 0: 154, 31.9%; Cluster 1: 329, 68.1%). Specifically, the cluster separation generated through this approach achieved a stability index of 0.957 for K = 2. Consistent with the established molecular signatures and clinical prognostic implications of the binary ccA/ccB classifications of ccRCC, the current work demonstrates a high degree of overlap between these two sets of classifications, where Cluster 0 can be mapped to the ccB archetype, and Cluster 1 corresponds to ccA. The original ccA/ccB classifier used an unsupervised gene expression consensus clustering approach, in which ccA tumors were associated with angiogenesis, hypoxia, and fatty acid metabolism gene programs, whereas ccB tumors showed enrichment for cell cycle genes and epithelial-mesenchymal transition (EMT) [17]. While the transcriptomic phenotypes of our identified clusters mirror the historically established ccA and ccB archetypes, the primary novelty of our study lies in the unmasking of the upstream regulatory architecture driving these expression states. Pure transcriptomic classifications, while prognostically valuable, are highly descriptive and often fail to provide actionable molecular targets. Unlike purely descriptive approaches or earlier multi-level genomic taxonomies [12], integrating RABIT and PARADIGM scores transitions our approach from identifying downstream phenotypes to uncovering their mechanistic origins. For instance, determining that the aggressive ccB-like phenotype (Cluster 0) is mechanistically dependent on the SREBF1 lipid-metabolic axis and E2F-driven cell cycle programs provides direct, targetable vulnerabilities (e.g., CDK4/6 inhibitors or lipid desaturation targeting) that standard RNA-seq clustering cannot reliably infer. Thus, our classification validates historical archetypes while simultaneously bridging the gap between descriptive subtyping and therapeutic intervention. Previous investigations employing pathway integrated analyses in ccRCC have shown that adding upstream pathway and immune context layers improves subtype stability and clinical prediction beyond transcriptomic classifications [11,13]. Importantly, in the present analysis, Cluster 0 is mutually exclusive with cell cycle programs (E2F Targets; NES = 2.140, FDR < 0.001; G2-M Checkpoint; NES = 1.548, FDR = 0.016), while Cluster 1 showed mutual exclusivity with stromal and angiogenic programs (Angiogenesis; NES = −1.816, FDR < 0.001; Inflammatory Response; NES = −1.623, FDR = 0.010; EMT; NES = −1.767, FDR = 0.003). This is consistent with the anti-correlated transcriptional polarities between proliferative and angiogenic gene programs observed across independent ccRCC cohorts [8].

The Proliferative/Metabolic phenotype exhibited by Cluster 0 represents a state associated with bioenergetic burden, resulting from the coordinated activation of cell cycle programs regulated by E2F and lipid metabolism regulated by SREBF1 [7,18]. Notable targets of the dominant E2F-regulated transcriptional circuits include AURKA, BIRC5, CCNB2, CDC25A, PLK1, and CDK1, a panel of genes encoding mitotic regulators, cyclins, and kinases, whose overexpression was correlated with increased pathological stage, poor molecular classification, and decreased survival in ccRCC [16]. Differential expression analysis identified 880 genes that were significantly differentially expressed, of which 86 were upregulated in Cluster 0, including the metabolic enzymes HAGHL and RHCG. RABIT-inferred SREBF1 (SREBP1) activity was the most significant TF distinguishing Cluster 0 from Cluster 1. SREBP1 is a master regulator of lipid desaturation whose activation is directly linked to tumor cell proliferation. It was found to be overexpressed in ccRCC cell lines and mechanistically linked to lipid desaturation through downstream NF-κB activation, with its inhibition significantly impairing ccRCC cell growth [18]. Moreover, enrichment of the Cholesterol homeostasis hallmark gene set in Cluster 0 further supports this hypothesis, as SREBF1 activity is part of a VHL-HIF-2α-MED15-SREBP1 signaling axis that mediates lipid desaturation and lipid accumulation in ccRCC [5]. The connection between E2F-dependent cell cycle transcription and SREBF1-dependent lipid synthesis is consistent with reports of direct E2F1 regulation of SREBF1, which drives ccRCC proliferation and metastasis through lipid metabolic reprogramming [7]. Therefore, the metabolic phenotype, characterized by overexpression of metabolic genes and increased SREBF1 activity, complemented by activation of E2F-dependent cell cycle transcription, establishes a fully functional circuit involving proliferation and metabolic pathways in this subtype. This phenotype is additionally corroborated by increased expression of mitochondrial respiratory chain genes and PARADIGM-inferred respiratory electron transport activity (t = 22.32, *p* = 6.26 × 10^−63^), allowing for sufficient energy production to meet the bioenergetic requirements of proliferation [12].

From a therapeutic perspective, Cluster 0 presents several exploitable vulnerabilities with immediate translational implications. Specifically, the co-activation of E2F-driven cell cycle programs and SREBF1-driven lipid metabolism suggests a dual proliferative/metabolic dependence in Cluster 0 tumors, amenable to pharmacological inhibition. Since Cluster 0 is characterized by a significantly inferior survival rate and lacks an angiogenesis-related gene expression pattern, VHL loss in these tumors is unlikely to drive angiogenic transformation of the tumor microenvironment. Therefore, ccRCC patients in Cluster 0 are expected to exhibit resistance to VEGFR-TKI monotherapy, as seen with analogous ccB subtypes, with a response rate of only 22% compared to 53% for ccA subtypes under sunitinib treatment [17,19]. As a consequence of this intrinsic resistance, the exploration of E2F-dependent cell cycle regulators, including CDK4/6 inhibitors (e.g., ongoing clinical trials combining CDK4/6 inhibitors with TKIs in RCC), as well as new treatments targeting SREBF1 and related lipid metabolic programs in Cluster 0, should be strongly considered [16,20]. This is supported by multiple preclinical studies suggesting that SREBF1 is a viable therapeutic target in ccRCC patients [18,20]. Finally, the presence of an active respiratory electron transport chain in Cluster 0 suggests that this subtype of ccRCC may be sensitive to electron transport chain inhibitors such as metformin or IACS-010759, which target high mitochondrial activity [21].

On the contrary, the Angiogenic/Inflammatory profile of Cluster 1 reflects the classical characteristics of VHL mutations [5,22]. In particular, the PARADIGM-inferred Notch signaling network stood out as the most prominently activated in Cluster 1. Notably, this network included endothelin and Notch receptor cleavage pathways, suggesting a mature Notch-driven vascular niche in ccRCC tumors. In ccRCC, Notch-DLL4 signaling drives tumor-associated vasculature remodeling and cancer stem cell maintenance, and its cooperation with VEGF-driven angiogenic programs contributes to the self-reinforcing nature of VHL-mutated tumors [22]. The prominent members of the angiogenesis hallmark in Cluster 1 are VEGFA, COL3A1, POSTN, and THBD, all well-known angiogenic factors regulated by hypoxia-inducible transcription factors. The most overrepresented gene in Cluster 1 was integrin alpha-8 (ITGA8), which encodes a matrix adhesion receptor that plays a crucial role in vascular smooth muscle and stromal niche formation. Meanwhile, the enrichment of Inflammatory response, TNF-α Signaling via NF-κB, allograft rejection, and IL-2/STAT5 Signaling hallmarks implied an immune-infiltrated tumor microenvironment in Cluster 1. The TF activity analysis demonstrated a highly active inflammatory environment driven by IKZF1 (lymphoid lineage transcription factor Ikaros), SPI1 (myeloid lineage transcription factor PU.1), and FOS (cytokine-activated inflammation-related transcription factor) [11].

The observed advantage in survival rate in Cluster 1 (HR = 0.70, 95% CI 0.51–0.96, *p* = 0.026) is likely attributable to two main mechanisms. Firstly, the extensive lymphoid (IKZF1-driven) and myeloid (SPI1-driven) infiltrates at the invasive margin suggest an intrinsic tumor microenvironment capable of mounting an innate anti-tumoral immune response, a fact confirmed by the external validation where Cluster 1 was found to have higher margin-to-center T cell ratio (*p* = 2.18 × 10^−2^) than Cluster 0. This specific spatial distribution indicates an “excluded” or “marginal” immune phenotype rather than a depleted one. Biologically, margin-enriched CD8+ infiltration is highly significant; it is consistent with a favorable response to ICIs, as margin-infiltrating T cells can be reinvigorated by anti-PD1 therapies to overcome spatial immune exclusion. Secondly, the fact that highly angiogenic and ccA-like tumors respond better to anti-angiogenic therapy helps to explain survival advantages in this subgroup [17,19]. In ccRCC, distinct, validated transcriptomic subgroups reveal an immune-silent but highly angiogenic tumor class with superior response to VEGFR-TKIs, alongside immunologically active tumor classes that benefit from immune checkpoint blockade [13]. The immune-inflammatory phenotype of Cluster 1, defined by Ikaros-driven lymphoid infiltrates and PU.1-driven myeloid infiltration, suggests that combining immunotherapy with TKIs could be a potentially successful treatment regimen [23].

Notably, RABIT-inferred TF activity profiles confirmed the differential immune/mutational characteristics of the two subtypes. In Cluster 1, the top three TFs were as follows: IKZF1 (Ikaros; t = −13.15, *p* = 1.81 × 10^−30^), SPI1 (PU.1; t = −13.12, *p* = 1.09 × 10^−30^), and FOS (t = −12.80, *p* = 2.30 × 10^−30^). IKZF1 governs the fate of the lymphoid lineage, promoting differentiation of T and B cells, consistent with the IL-2/STAT5, allograft rejection, and interferon-γ hallmarks, implying the predominance of the lymphoid response. SPI1 (PU.1) is a master transcription factor of the myeloid lineage and drives the development of macrophages, dendritic cells, and monocytes; thus, it explains a high degree of macrophage involvement, previously shown to determine survival in ccRCC [11]. Finally, FOS, a member of the AP-1 family of transcription factors, participates in cytokine-induced inflammatory transcription and thus aligns with the TNF-α and NF-κB pathway enrichments. NF-κB-driven inflammatory signals are often linked to immune evasion and disease progression in ccRCC [14].

In Cluster 0, SREBF1 and SP1 (t = 9.25, *p* = 5.32 × 10^−18^) dominated the TF profile. The latter controls the transcription of HIF-target genes, cell-cycle genes, and mitochondrial biogenesis genes, whereas high ZEB1 activity (t = 8.30) suggests epithelial-to-mesenchymal transition and proliferation in this subtype. The top PARADIGM features of the Cluster 0 phenotype, including Respiratory electron transport (t = 22.32, *p* = 6.26 × 10^−63^) and Early elongation complex formation (t = 22.41, *p* = 2.59 × 10^−61^), suggest a high bioenergetic burden associated with proliferation [12]. Overall, the concordance of results from different analyses strengthens the interpretation of subtypes and confirms the previously established importance of NF-κB, PXR, and SREBP1 activity in determining prognosis in ccRCC [14,15].

To develop the above findings into a clinically usable classification framework, the Random Forest model was constructed on the TCGA discovery set using the top 30 Recursive Feature Elimination-selected features. Methodologically, one of the unique features of the present Random Forest model was that the optimization criterion for model parameters targeted survival stratification, aiming to obtain the lowest Log-Rank *p*-value. By design, this approach’s objective matched the goal of stratifying ccRCC patients by outcome, making it distinct from other supervised classification algorithms used in machine learning applications [11,13]. The resulting prediction accurately stratified the external validation cohort by survival rate (Log-Rank *p* = 1.66 × 10^−2^), confirming that the biological signal remained intact even after quantile normalization to account for inter-platform expression variability. Moreover, external validation confirmed significantly higher tumor margin-to-center CD8 ratios in Cluster 1 (*p* = 2.18 × 10^−2^), in agreement with the predictions. Neither Objective Response Rate, nor ImmunoPhenotype, Extreme Responder status, nor Clinical Benefit differed between the two predicted subgroups.

However, several shortcomings in this study warrant note. First, the bulk RNA-seq technique used does not capture the heterogeneity of intratumoral immune cells, stroma, and tumor cells [24]. Consequently, we were unable to investigate cellular distribution or functional differences at single-cell resolution. This inherently limits our ability to make definitive subtype-specific therapeutic predictions. Recent studies have demonstrated that resolving tumor microenvironment heterogeneity at the single-cell level is critical for accurately predicting responses to immunotherapies [25,26]. Second, the computational-based PARADIGM and RABIT scores need further proteomic and biochemical verification [12]. Third, the retrospective nature and the surgical cases of the majority of TCGA patients introduce inherent biases into the results [5]. Fourth, OS is a composite outcome, combining cancer-specific and non-cancer mortality rates; therefore, future studies should aim to examine the survival benefit from subtype-based treatment using disease-specific or progression-free survival [27]. Finally, survival-guided Random Forest is vulnerable to overfitting and needs prospective validation in a biomarker study. To address these limitations, future research should focus on using single-cell and high-resolution spatial transcriptomics to understand the composition, cellular architecture, and immune-vascular crosstalk specific to each subgroup [25,26,28]. Mechanistically, perturbation experiments using CRISPR-Cas9 knockout techniques will be necessary to elucidate the roles of these master regulators, specifically IKZF1 in Cluster 1 and SREBF1 in Cluster 0 [29]. In clinical practice, prospective biomarker studies must test the potential utility of these subgroups in predicting the efficacy of VEGFR-TKI monotherapy compared with current TKI-ICI regimens [23]. Ultimately, integrating liquid biopsy platforms (e.g., ctDNA, cfRNA) could enable longitudinal assessment of subtype stability during disease progression and provide a valuable clinical tool for precision oncology of ccRCC [30].

## 5. Conclusions

The current investigation used a pathway-integrated consensus clustering method combining mRNA expression, PARADIGM pathway activity, and RABIT TF activity to categorize the 483 ccRCC samples from TCGA-KIRC into two distinct subtypes with clear prognostic and mechanistic differences, consistent with the classical ccA/ccB dichotomy. The Proliferative/Metabolic cluster (Cluster 0), which encompasses about 32% of all samples, reflects a very aggressive, ccB-type pattern characterized by E2F-driven cell cycle activation and reprogrammed lipid metabolism mediated by SREBF1. Cluster 0 demonstrates increased mitochondrial bioenergetic potential, inferior overall survival, and most likely, intrinsic resistance to VEGFR-TKI-based monotherapy. Thus, alternative treatment approaches targeting cell cycle regulation or lipid metabolism may be beneficial for patients with Cluster 0 tumors. On the other hand, the Angiogenic/Inflammatory cluster (Cluster 1), which represents 68% of all tumors, correlates with a ccA-type tumor characterized by classic HIF-driven Notch-dependent angiogenesis and a rich immune microenvironment maintained by IKZF1 and SPI1. Importantly, this group shows a significant survival benefit (HR = 0.70) and externally validated enrichment of CD8+ T cells along the invasive margins, suggesting that Cluster 1 is an ideal candidate for first-line TKI-based therapy or for combinations of TKIs and ICIs. Survival-guided Random Forest classification algorithm verified the biological consistency and clinical relevance of these clusters in an independent dataset. Considering the integration of higher-order biological processes, the presented methodology goes beyond standard transcriptomics to provide actionable biological insight into molecular heterogeneity and, consequently, precision medicine in ccRCC.

## Figures and Tables

**Table 1:** Baseline characteristics of the discovery and validation cohorts.

Characteristic	TCGA Discovery (*n* = 483)	CheckMate Validation (*n* = 225)
No. of patients, *n*	483	225
Age, years, median [IQR]	61 (52–70)	62 (54–68)
Sex, *n* (%)	—	—
Male	312 (64.6%)	164 (72.9%)
Female	171 (35.4%)	61 (27.1%)
Pathologic stage, *n* (%)		—
Stage I	230 (47.6%)	—
Stage II	53 (11.0%)	—
Stage III	118 (24.4%)	—
Stage IV	82 (17.0%)	—
Histologic grade, *n* (%)		—
G1	10 (2.1%)	—
G2	201 (41.6%)	—
G3	192 (39.8%)	—
G4	73 (15.1%)	—
Unknown/GX	7 (1.4%)	—
Death events, *n* (%)	165 (34.2%)	173 (76.9%)
Median overall survival, months (95% CI)	90.4 (73.2–NR)	22.2 (19.5–25.9)
Follow-up time, months, median (IQR)	38 (17–63)	21 (9–46)
MSKCC risk group, *n* (%)	—	
Intermediate	—	104 (46.2%)
Favorable	—	73 (32.4%)
Poor	—	48 (21.3%)
IMDC risk group, *n* (%)	—	
Intermediate	—	110 (48.9%)
Poor	—	40 (17.8%)
Favorable	—	28 (12.4%)
Not Reported	—	2 (0.9%)
Treatment arm, *n* (%)	—	
Nivolumab	—	133 (59.1%)
Everolimus	—	92 (40.9%)

OS, overall survival; IQR, interquartile range; CI, confidence interval; NR, not reached; MSKCC, Memorial Sloan Kettering Cancer Center; IMDC, International Metastatic RCC Database Consortium.

**Table 2:** Multivariate Cox proportional hazards regression analysis of overall survival.

Variable	HR	95% CI	*p*-Value
Molecular Cluster (C1 vs. C0)	0.70	0.51–0.96	0.026
Age (standardized)	1.41	1.18–1.69	<0.001
Tumor Stage (per unit)	1.91	1.66–2.20	<0.001
Gender (Female vs. Male)	0.95	0.69–1.32	0.774

HR, hazard ratio; CI, confidence interval. Reference group: Cluster 0. Adjusted for age (standardized), tumor stage (ordinal 1–4), and gender.

**Table 3:** Internal validation performance of the Random Forest classifier.

Metric	Value (95% CI)
Accuracy	0.934 (0.927–0.941)
AUROC	0.984 (0.976–0.992)
Sensitivity	0.951 (0.930–0.971)
Specificity	0.890 (0.845–0.936)
Out-of-bag (OOB) accuracy	0.942
Out-of-bag (OOB) error	0.058

Metrics from stratified 5-fold cross-validation on the TCGA discovery cohort (mean with 95% CI across folds). OOB error is an independent held-out estimate not used in survival-based model selection.

## Data Availability

All relevant data are included within the manuscript and its supplementary files. The raw datasets analyzed during the current study are publicly available in the TCGA repository. The computational pipelines utilized standard open-source Python libraries. Custom scripts and intermediate data matrices are available from the corresponding author upon reasonable request.

## Supplementary Materials

The following supporting information can be downloaded at: https://www.mdpi.com/article/10.3390/cancers18213485/s1, Table S1: Consensus clustering stability across candidate cluster numbers; Table S2: Complete GSEA Hallmark pathway results; Table S3: Top differentially expressed genes (FDR < 0.05, |Log2FC| > 1.0); Table S4: External cohort clinical association results; Figure S1: Individual GSEA enrichment plot for the E2F Targets Hallmark gene set; Figure S2: Individual GSEA enrichment plot for the G2-M Checkpoint Hallmark gene set; Figure S3: Individual GSEA enrichment plot for the Cholesterol Homeostasis Hallmark gene set; Figure S4: Individual GSEA enrichment plot for the Angiogenesis Hallmark gene set; Figure S5: Individual GSEA enrichment plot for the Epithelial Mesenchymal Transition Hallmark gene set; Figure S6: Individual GSEA enrichment plot for the Inflammatory Response Hallmark gene set; Figure S7: Individual GSEA enrichment plot for the TNF-alpha Signaling via NF-κB Hallmark gene set; Figure S8: Individual GSEA enrichment plot for the Allograft Rejection Hallmark gene set; Figure S9: Individual GSEA enrichment plot for the IL-2/STAT5 Signaling Hallmark gene set; Figure S10: Individual GSEA enrichment plot for the Mitotic Spindle Hallmark gene set; Figure S11: Individual GSEA enrichment plot for the KRAS Signaling Up Hallmark gene set; Figure S12: Individual GSEA enrichment plot for the Apical Junction Hallmark gene set; Figure S13: Individual GSEA enrichment plot for the Hedgehog Signaling Hallmark gene set; Figure S14: Individual GSEA enrichment plot for the TGF-beta Signaling Hallmark gene set; Figure S15: Individual GSEA enrichment plot for the Notch Signaling Hallmark gene set; Figure S16: Individual GSEA enrichment plot for the Wnt-beta Catenin Signaling Hallmark gene set; Figure S17: Individual GSEA enrichment plot for the PI3K/AKT/mTOR Signaling Hallmark gene set; Figure S18: Individual GSEA enrichment plot for the Myogenesis Hallmark gene set; Figure S19: Individual GSEA enrichment plot for the Interferon Gamma Response Hallmark gene set; Figure S20: Individual GSEA enrichment plot for the UV Response Dn Hallmark gene set; Figure S21: Consensus Cumulative Distribution Function (CDF) plots for K = 2 to K = 6.

## Abbreviations

The following abbreviations are used in this manuscript:

ccA	Clear cell A
ccB	Clear cell B
ccRCC	Clear cell renal cell carcinoma
cfRNA	Cell-free Ribonucleic acid
CI	Confidence Interval
CRISPR	Clustered Regularly Interspaced Short Palindromic Repeats
ctDNA	Circulating tumor Deoxyribonucleic acid
EMT	Epithelial-Mesenchymal Transition
FDR	False Discovery Rate
GSEA	Gene Set Enrichment Analysis
HIF	Hypoxia-Inducible Factor
HR	Hazard Ratio
ICIs	Immune Checkpoint Inhibitors
IFN	Interferon
IL-2	Interleukin-2
KIRC	Kidney Renal Clear Cell Carcinoma
Log2FC	Log2 Fold Change
MSigDB	Molecular Signatures Database
NES	Normalized Enrichment Score
NF-κB	Nuclear Factor kappa-light-chain-enhancer of activated B cells
ORR	Objective Response Rate
OS	Overall Survival
PXR	Pregnane X Receptor
QRDI	Qatar Research Development and Innovation Council
RFE	Recursive Feature Elimination
TC	Tumor Center
TCGA	The Cancer Genome Atlas
TF	Transcription Factor
TKIs	Tyrosine Kinase Inhibitors
TM	Tumor Margin
TNF-α	Tumor Necrosis Factor alpha
VEGF	Vascular Endothelial Growth Factor
VEGFR	Vascular Endothelial Growth Factor Receptor
VHL	Von Hippel-Lindau
