# Supplementary material for: Integrated Transcriptomic and Regulatory Network Analysis Reveals Distinct Molecular Subtypes with Prognostic Significance in Clear Cell Renal Cell Carcinoma
Source: Cancers (Basel). 2026 Oct 29;18(21):3485. doi: 10.3390/cancers18213485 (PMC13762045; doi:10.3390/cancers18213485)
Supplement: Supplementary file 1 [file cancers-18-03485-s001.zip › cancers-4361471-supplementary.pdf]

Supplementary Material

Integrated Transcriptomic and Regulatory Network Analysis Reveals Distinct Molecular Subtypes with Prognostic Significance in Kidney Renal Clear Cell Carcinoma

Table S1. Consensus clustering stability across candidate cluster numbers.
CDF AUC, area under the consensus cumulative distribution function; stability, silhouette score on the precomputed consensus distance. K = 2 was selected on maximal stability. Unsupervised MeanShift concordance with the K = 2 solution: ARI = 0.429. External validation (CheckMate, n = 225) log-rank p = 0.090.

Cluster number (K)	CDF AUC	Stability (silhouette)
K = 2 (selected)	0.283	0.947
K = 3	0.363	0.923
K = 4	0.472	0.691
K = 5	0.489	0.603
K = 6	0.528	0.445

Table S2. Complete GSEA Hallmark Pathway Results

Gene Set Enrichment Analysis (GSEA) was performed using gseapy with MSigDB Hallmark 2020 gene sets. Genes were ranked by Welch's t-statistic from the Cluster 0 vs. Cluster 1 differential expression analysis. Sign convention: positive NES indicates enrichment in Cluster 0 (Proliferative/Metabolic); negative NES indicates enrichment in Cluster 1 (Angiogenic/Inflammatory). All pathways with FDR q-value <0.10 are reported. Significance codes: *** FDR<0.001; ** FDR<0.01; * FDR<0.05; — FDR 0.05–0.10. Permutations: 100.

Hallmark Pathway	Enriched In	NES	FDR q-val	Significance
E2F Targets	Cluster 0	2.140	<0.001	***
Cholesterol Homeostasis	Cluster 0	1.645	0.024	*
G2-M Checkpoint	Cluster 0	1.548	0.016	*
UV Response Dn	Cluster 1	−1.847	<0.001	***
Angiogenesis	Cluster 1	−1.816	<0.001	***
Epithelial Mesenchymal Transition	Cluster 1	−1.767	0.003	**
Mitotic Spindle	Cluster 1	−1.673	0.008	**
Inflammatory Response	Cluster 1	−1.623	0.010	*
KRAS Signaling Up	Cluster 1	−1.598	0.011	*
TNF-alpha Signaling via NF-κB	Cluster 1	−1.578	0.015	*
Apical Junction	Cluster 1	−1.493	0.036	*
IL-2/STAT5 Signaling	Cluster 1	−1.468	0.046	*
Allograft Rejection	Cluster 1	−1.463	0.046	*
Hedgehog Signaling	Cluster 1	−1.463	0.042	*
TGF-beta Signaling	Cluster 1	−1.447	0.051	—
PI3K/AKT/mTOR Signaling	Cluster 1	−1.399	0.082	—

Table S3. Top Differentially Expressed Genes (FDR<0.05, |Log2FC|>1.0)

Differential gene expression was assessed using Welch's t-test. Multiple hypothesis correction applied using the Benjamini-Hochberg FDR procedure. Inclusion criteria: FDR<0.05 AND |Log2FC|>1.0. Log2FC positive values indicate upregulation in Cluster 1; negative values indicate upregulation in Cluster 0. For COX6A1, TLCD1, UQCRQ, NDUFS8, and MRPL41, exact Log2FC values were confirmed to exceed the |1.0| threshold in the volcano plot; precise values available from the complete differential expression file (04_Differential_Expression.csv). A total of 880 significant DEGs were identified: 794 upregulated in Cluster 1, 86 upregulated in Cluster 0.

Gene	Log2FC	FDR	Biological Role	Cluster
ITGA8	2.81	7.42×10^{-32}	Integrin α8; matrix adhesion/vascular niche	Cluster 1↑
SLC6A3	2.43	1.28×10^{-8}	Dopamine transporter; stromal context	Cluster 1↑
ZNF366	2.30	3.86×10^{-43}	Zinc finger protein; transcriptional regulation	Cluster 1↑
C14orf49	2.22	<0.05	Uncharacterized; stromal context	Cluster 1↑
HEG1	2.10	<0.05	Heart development EG homolog; endothelial	Cluster 1↑
CD93	2.00	<0.05	C-type lectin domain; endothelial/angiogenic	Cluster 1↑
ITGA9	1.90	<0.05	Integrin α9; cell adhesion receptor	Cluster 1↑
LOC100271831	−2.26	6.58×10^{-15}	Uncharacterized lncRNA/pseudogene	Cluster 0↑
HAGHL	−2.05	5.49×10^{-19}	Haloacid dehalogenase-like hydrolase; metabolic	Cluster 0↑
RHCG	−1.77	2.89×10^{-6}	Rh family C glycoprotein; ammonium transport	Cluster 0↑
COX6A1	<−1.0	<0.05	Cytochrome c oxidase subunit 6A1; Complex IV	Cluster 0↑
NDUFS8	<−1.0	<0.05	NADH:ubiquinone oxidoreductase; Complex I	Cluster 0↑
UQCRQ	<−1.0	<0.05	Ubiquinol-cytochrome c reductase; Complex III	Cluster 0↑
TLCD1	<−1.0	<0.05	TLC domain-containing protein 1; lipid metabolism	Cluster 0↑
MRPL41	<−1.0	<0.05	Mitochondrial ribosomal protein L41	Cluster 0↑

Table S4. External Cohort Clinical Association Results

Statistical tests were selected based on variable type and distribution: Mann-Whitney U test for continuous immune quantification variables; Chi-square test for categorical variables with expected frequencies ≥5; Fisher's Exact Test automatically applied when expected cell frequencies <5. Significance threshold: p<0.05. NS, not statistically significant. Note: the limited sample size and heterogeneity of treatment regimens in the external validation cohort may have reduced statistical power for detecting subtype-associated clinical differences.

Variable	Statistical Test	p-value	Significance	Interpretation
TC_CD8 (Tumor Center CD8+)	Mann-Whitney U	3.91×10^{-2}	*	Significantly higher in Cluster 1
TM_TC_Ratio (Margin:Center CD8)	Mann-Whitney U	2.18×10^{-2}	*	Significantly higher in Cluster 1
TC_CD8_Density	Mann-Whitney U	7.73×10^{-2}	Trend	Trend toward higher in Cluster 1
ORR (Objective Response Rate)	Chi-square/Fisher	0.383	NS	No significant difference
ImmunoPhenotype Classification	Chi-square	0.329	NS	No significant difference
Extreme Responder Status	Fisher's Exact	0.490	NS	No significant difference
Clinical Benefit	Chi-square/Fisher	0.457	NS	No significant difference

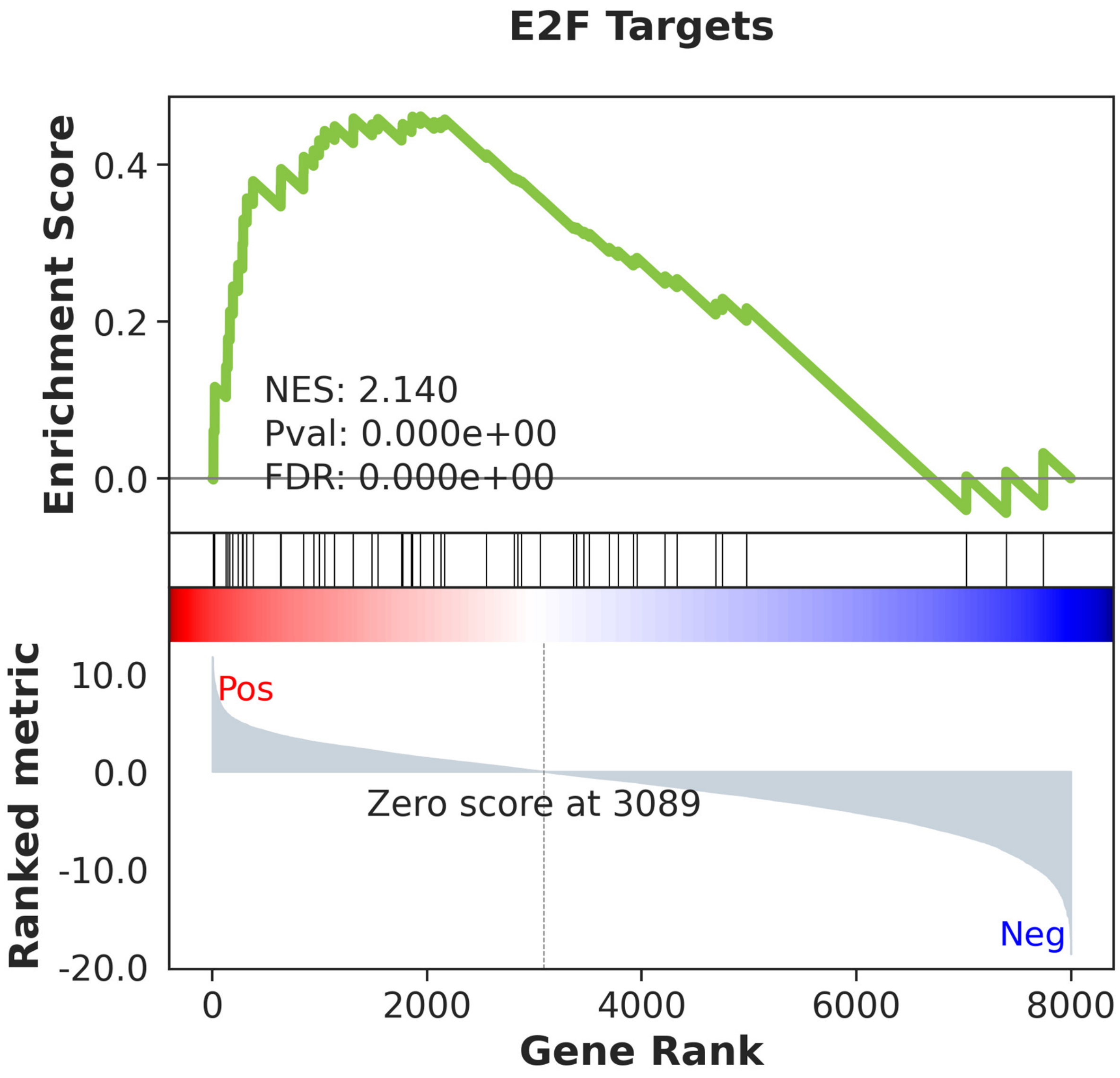

Figure S1. Individual GSEA enrichment plot for the E2F Targets Hallmark gene set. The plot shows three panels: (1) the running enrichment score (RES) curve, which peaks or troughs at the maximum enrichment score (ES); (2) vertical bars marking the positions of gene set members in the ranked gene list; and (3) a heat map of the ranked gene metric (Welch's t-statistic) along the list. The E2F Targets pathway was significantly

enriched in Cluster 0 (Proliferative/Metabolic) (NES=2.140, NOM p-value=<0.001, FDR q-value=<0.001). Positive NES indicates that the leading-edge genes are concentrated at the top of the ranked list, corresponding to genes with higher expression in Cluster 0.

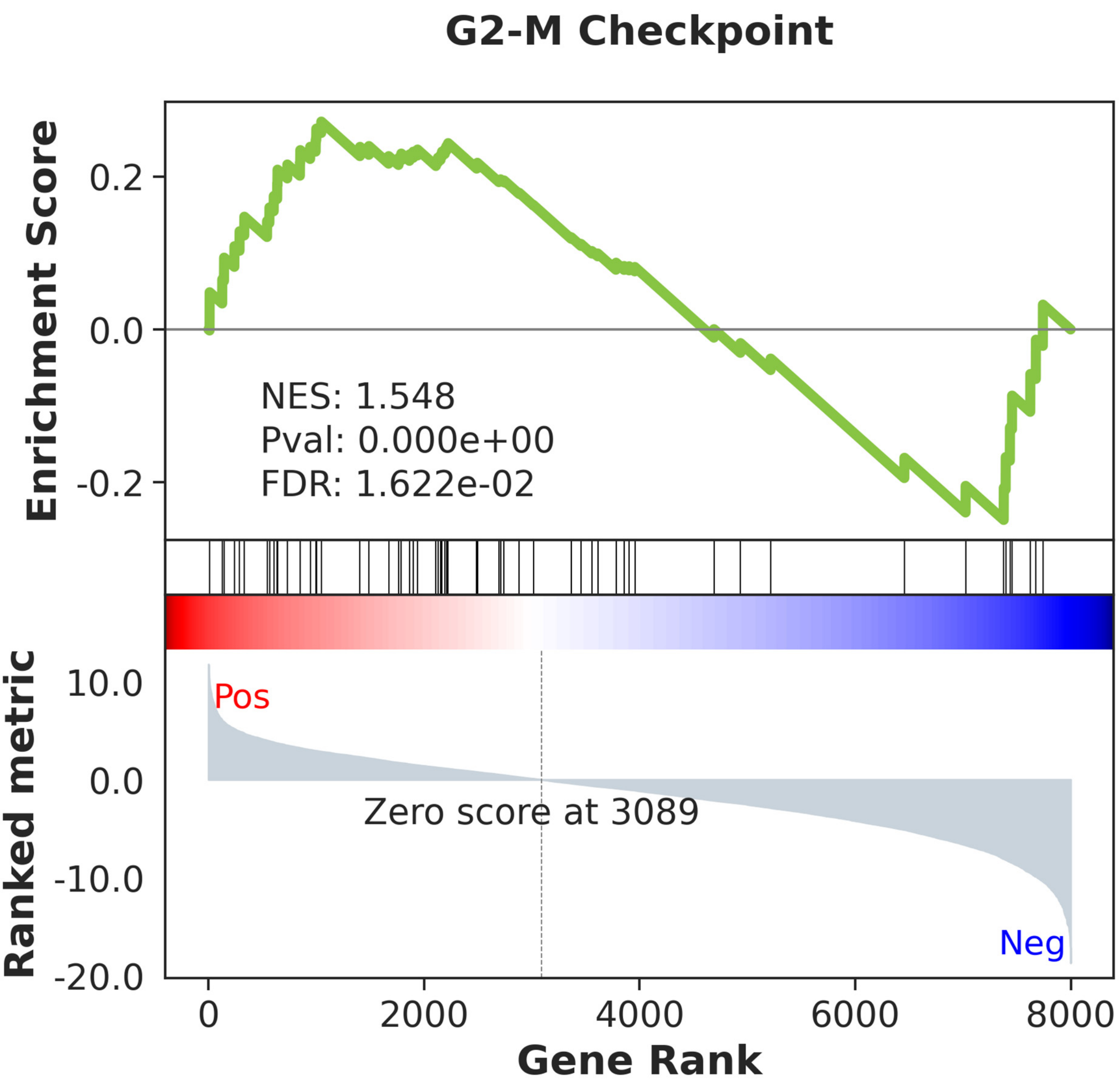

Figure S2. Individual GSEA enrichment plot for the G2-M Checkpoint Hallmark gene set. The plot shows three panels: (1) the running enrichment score (RES) curve, which peaks or troughs at the maximum enrichment score (ES); (2) vertical bars marking the positions of gene set members in the ranked gene list; and (3) a heat map of the ranked gene metric (Welch's t-statistic) along the list. The G2-M Checkpoint pathway was significantly enriched in Cluster 0 (Proliferative/Metabolic) (NES=1.548, NOM p-value=<0.001, FDR q-value=0.016). Positive NES indicates that the leading-edge genes are concentrated at the top of the ranked list, corresponding to genes with higher expression in Cluster 0.

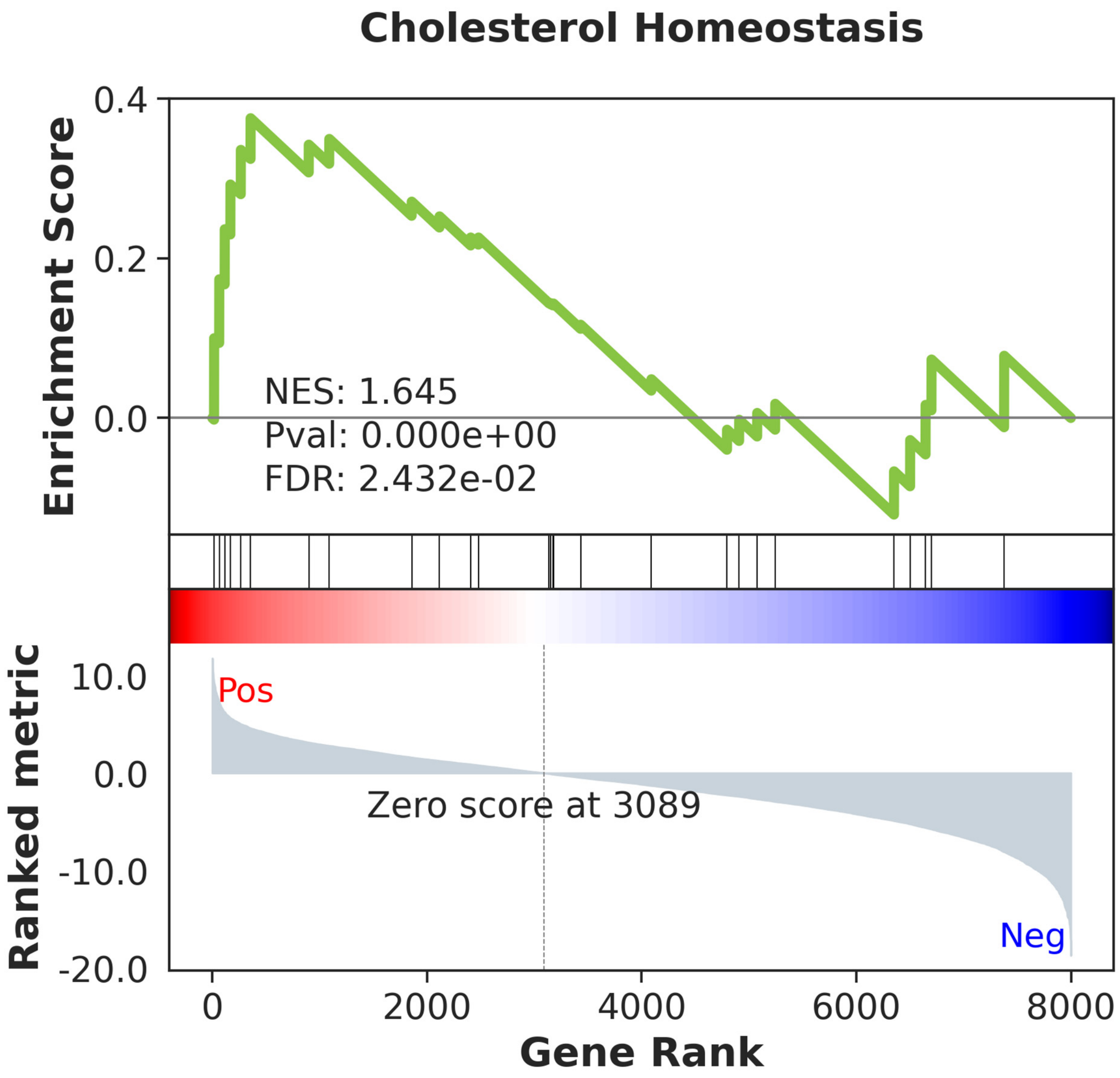

Figure S3. Individual GSEA enrichment plot for the Cholesterol Homeostasis Hallmark gene set. The plot shows three panels: (1) the running enrichment score (RES) curve, which peaks or troughs at the maximum enrichment score (ES); (2) vertical bars marking the positions of gene set members in the ranked gene list; and (3) a heat map of the ranked gene metric (Welch's t-statistic) along the list. The Cholesterol Homeostasis pathway was significantly enriched in Cluster 0 (Proliferative/Metabolic) (NES=1.645, NOM p-value=<0.001, FDR q-value=0.024). Positive NES indicates that the leading-edge genes are concentrated at the top of the ranked list, corresponding to genes with higher expression in Cluster 0.

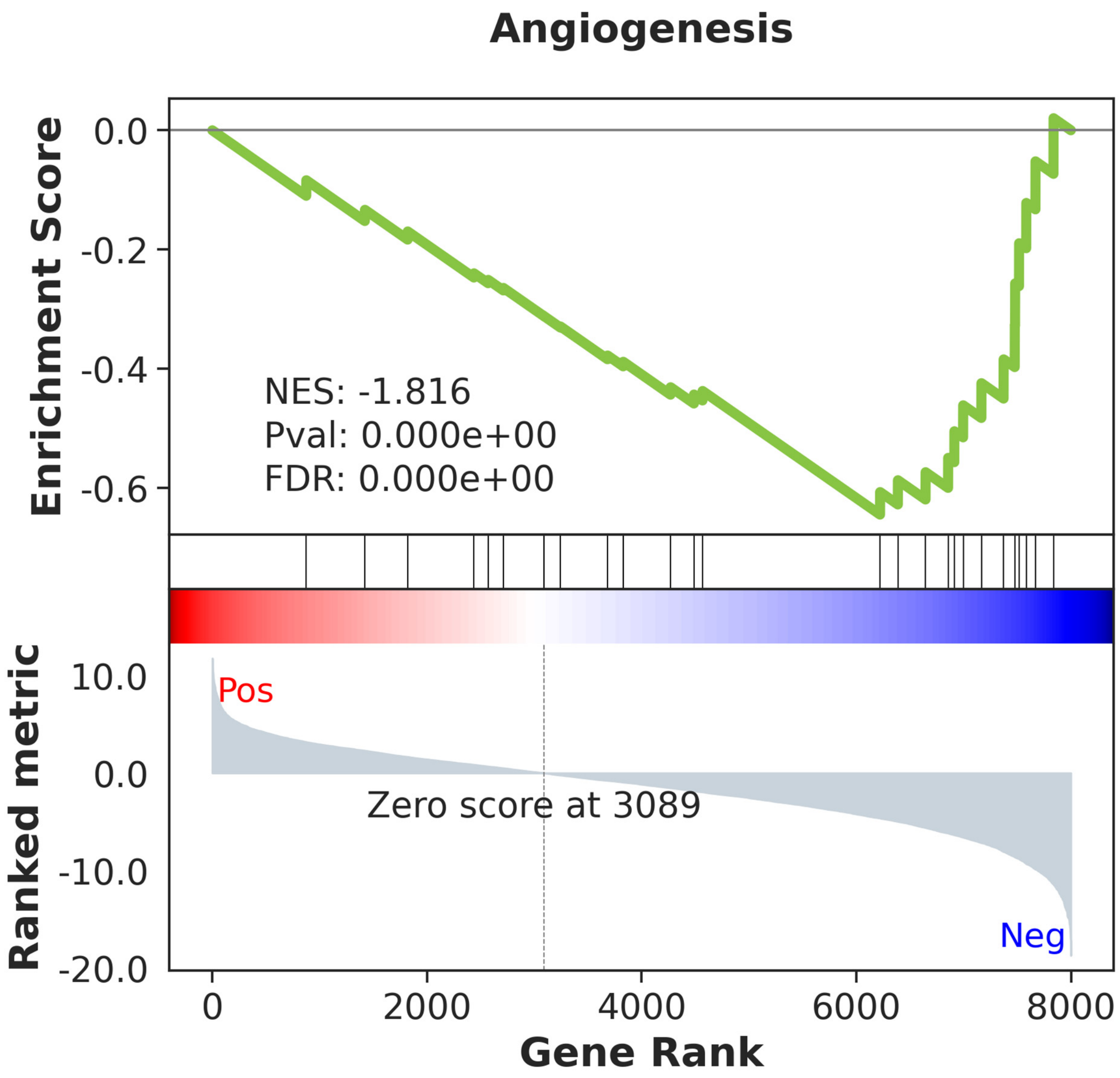

Figure S4. Individual GSEA enrichment plot for the Angiogenesis Hallmark gene set. The plot shows three panels: (1) the running enrichment score (RES) curve, which peaks or troughs at the maximum enrichment score (ES); (2) vertical bars marking the positions of gene set members in the ranked gene list; and (3) a heat map of the ranked gene metric (Welch's t-statistic) along the list. The Angiogenesis pathway was significantly enriched in Cluster 1 (Angiogenic/Inflammatory) (NES=−1.816, NOM p-value=<0.001, FDR q-value=<0.001). Negative NES indicates that the leading-edge genes driving this enrichment are concentrated at the bottom of the ranked list, corresponding to genes with higher expression in Cluster 1.

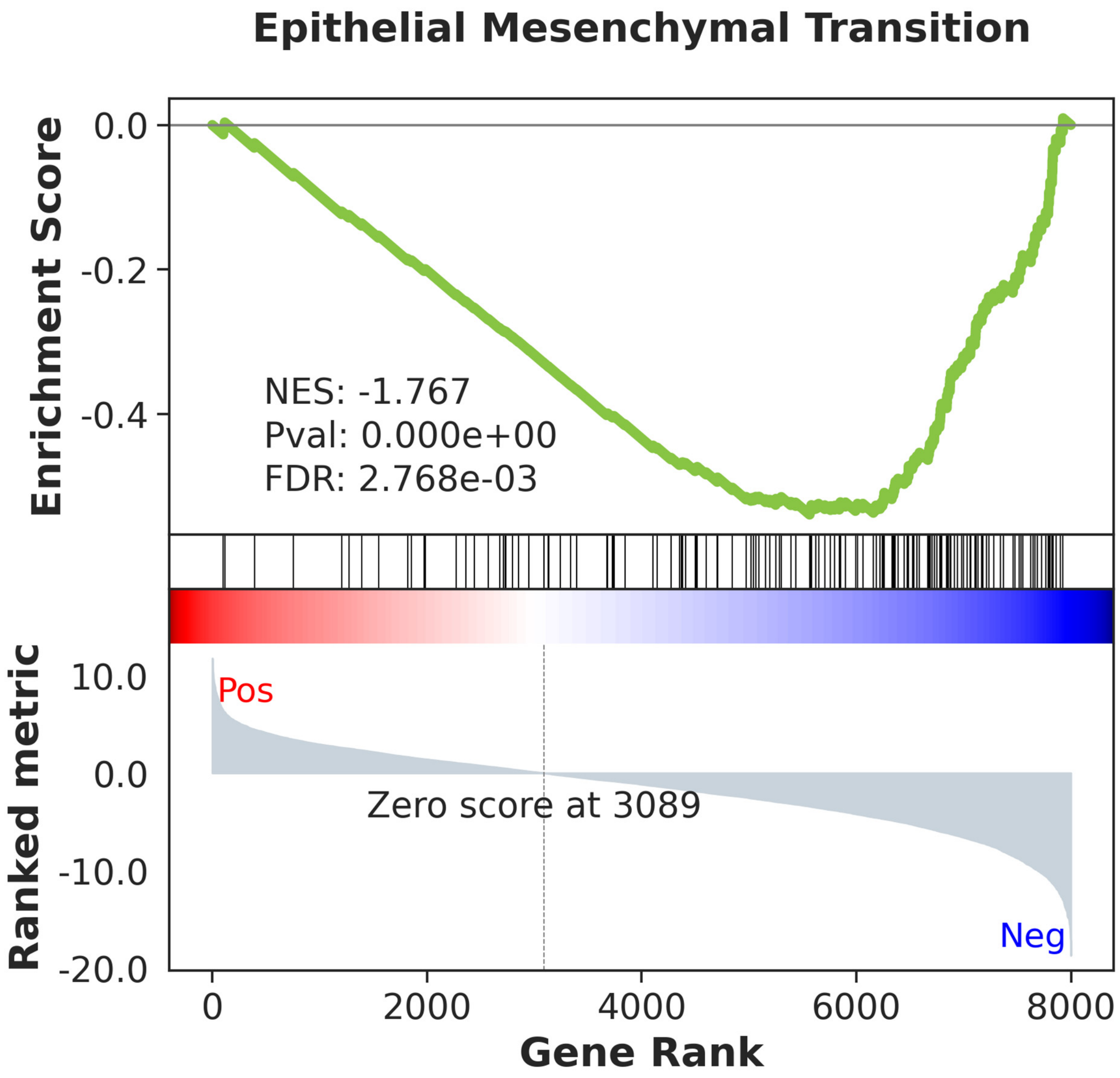

Figure S5. Individual GSEA enrichment plot for the Epithelial Mesenchymal Transition Hallmark gene set. The plot shows three panels: (1) the running enrichment score (RES) curve, which peaks or troughs at the maximum enrichment score (ES); (2) vertical bars marking the positions of gene set members in the ranked gene list; and (3) a heat map of the ranked gene metric (Welch's t-statistic) along the list. The Epithelial Mesenchymal Transition pathway was significantly enriched in Cluster 1 (Angiogenic/Inflammatory) (NES=−1.767, NOM p-value=<0.001, FDR q-value=0.003). Negative NES indicates that the leading-edge genes driving this enrichment are concentrated at the bottom of the ranked list, corresponding to genes with higher expression in Cluster 1.

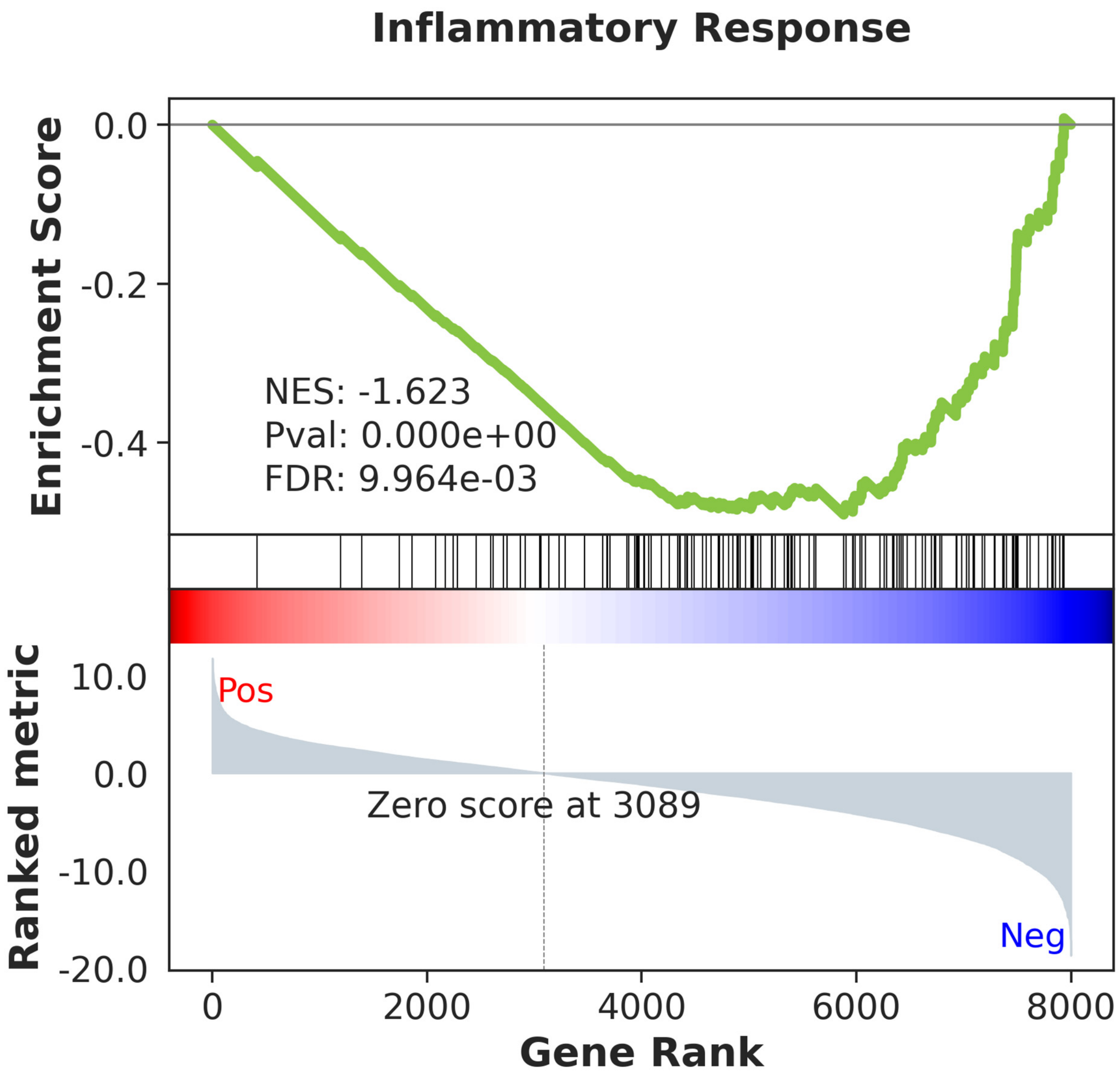

Figure S6. Individual GSEA enrichment plot for the Inflammatory Response Hallmark gene set. The plot shows three panels: (1) the running enrichment score (RES) curve, which peaks or troughs at the maximum enrichment score (ES); (2) vertical bars marking the positions of gene set members in the ranked gene list; and (3) a heat map of the ranked gene metric (Welch's t-statistic) along the list. The Inflammatory Response pathway was significantly enriched in Cluster 1 (Angiogenic/Inflammatory) (NES=−1.623, NOM p-value=<0.001, FDR q-value=0.010). Negative NES indicates that the leading-edge genes driving this enrichment are concentrated at the bottom of the ranked list, corresponding to genes with higher expression in Cluster 1.

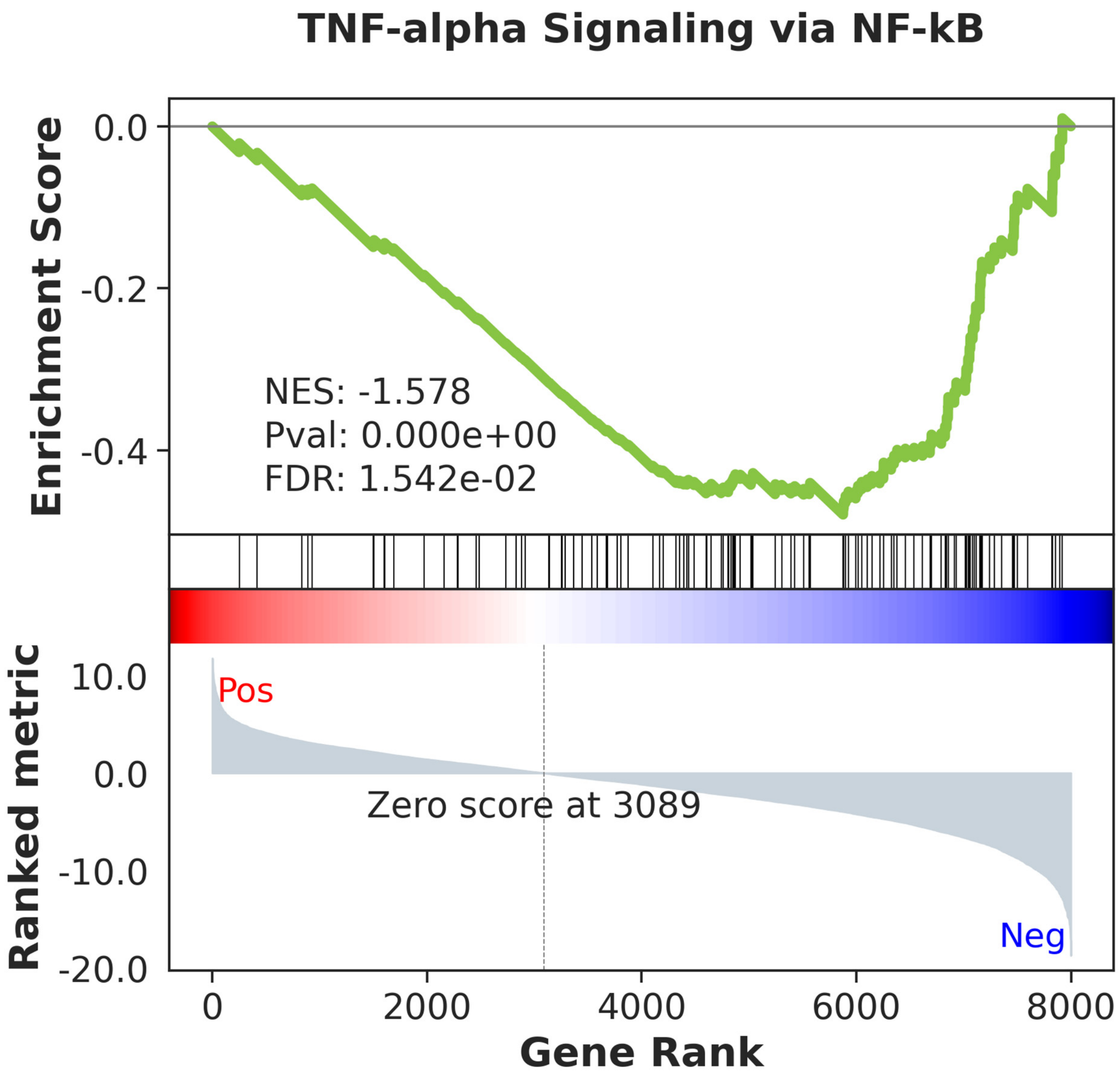

Figure S7. Individual GSEA enrichment plot for the TNF-alpha Signaling via NF-κB Hallmark gene set. The plot shows three panels: (1) the running enrichment score (RES) curve, which peaks or troughs at the maximum enrichment score (ES); (2) vertical bars marking the positions of gene set members in the ranked gene list; and (3) a heat map of the ranked gene metric (Welch's t-statistic) along the list. The TNF-alpha Signaling via NF-κB pathway was significantly enriched in Cluster 1 (Angiogenic/Inflammatory) (NES=−1.578, NOM p-value=<0.001, FDR q-value=0.015). Negative NES indicates that the leading-edge genes driving this enrichment are concentrated at the bottom of the ranked list, corresponding to genes with higher expression in Cluster 1.

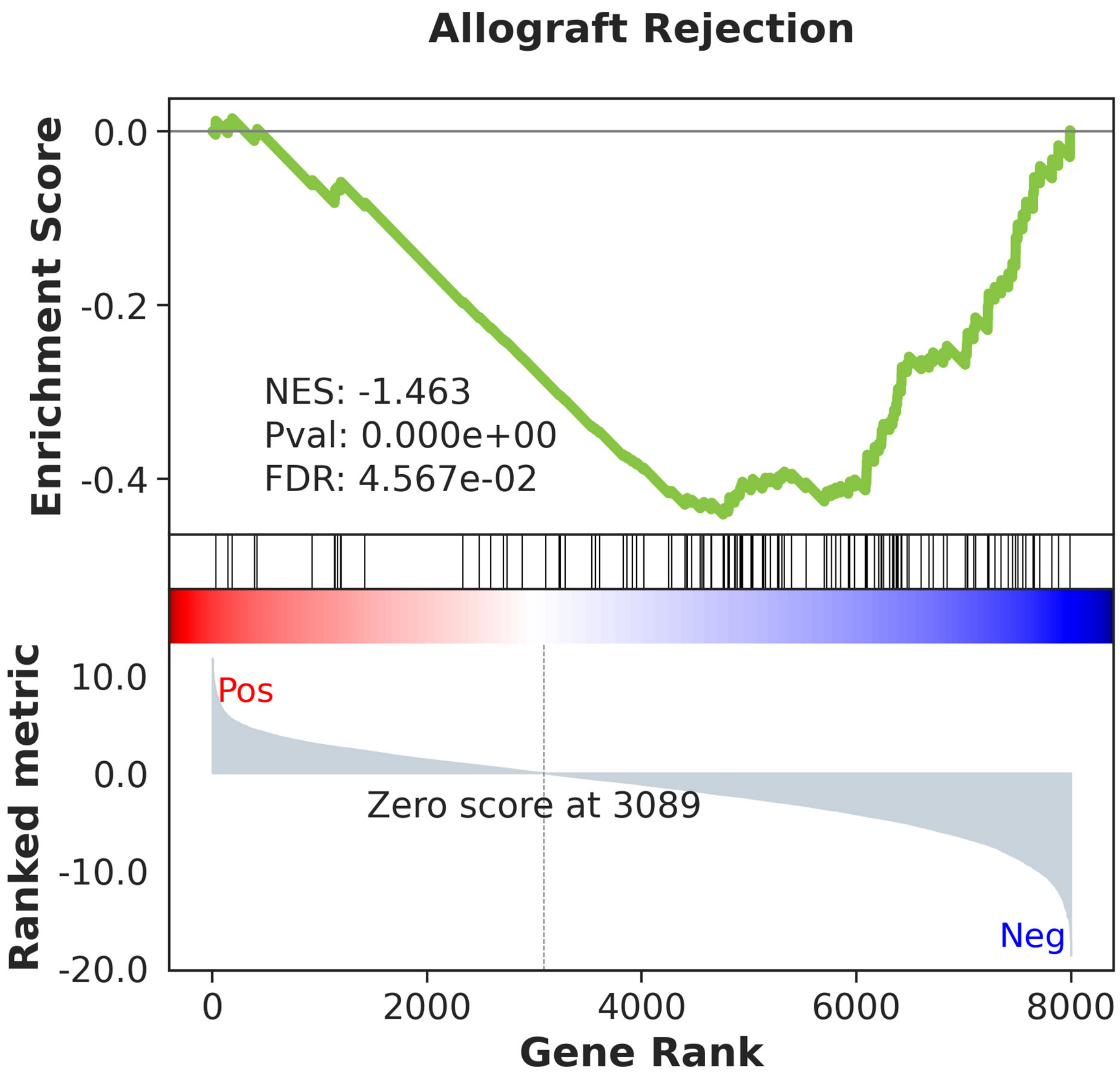

Figure S8. Individual GSEA enrichment plot for the Allograft Rejection Hallmark gene set. The plot shows three panels: (1) the running enrichment score (RES) curve, which peaks or troughs at the maximum enrichment score (ES); (2) vertical bars marking the positions of gene set members in the ranked gene list; and (3) a heat map of the ranked gene metric (Welch's t-statistic) along the list. The Allograft Rejection pathway was significantly enriched in Cluster 1 (Angiogenic/Inflammatory) (NES=−1.463, NOM p-value=<0.001, FDR q-value=0.046). Negative NES indicates that the leading-edge genes driving this enrichment are concentrated at the bottom of the ranked list, corresponding to genes with higher expression in Cluster 1.

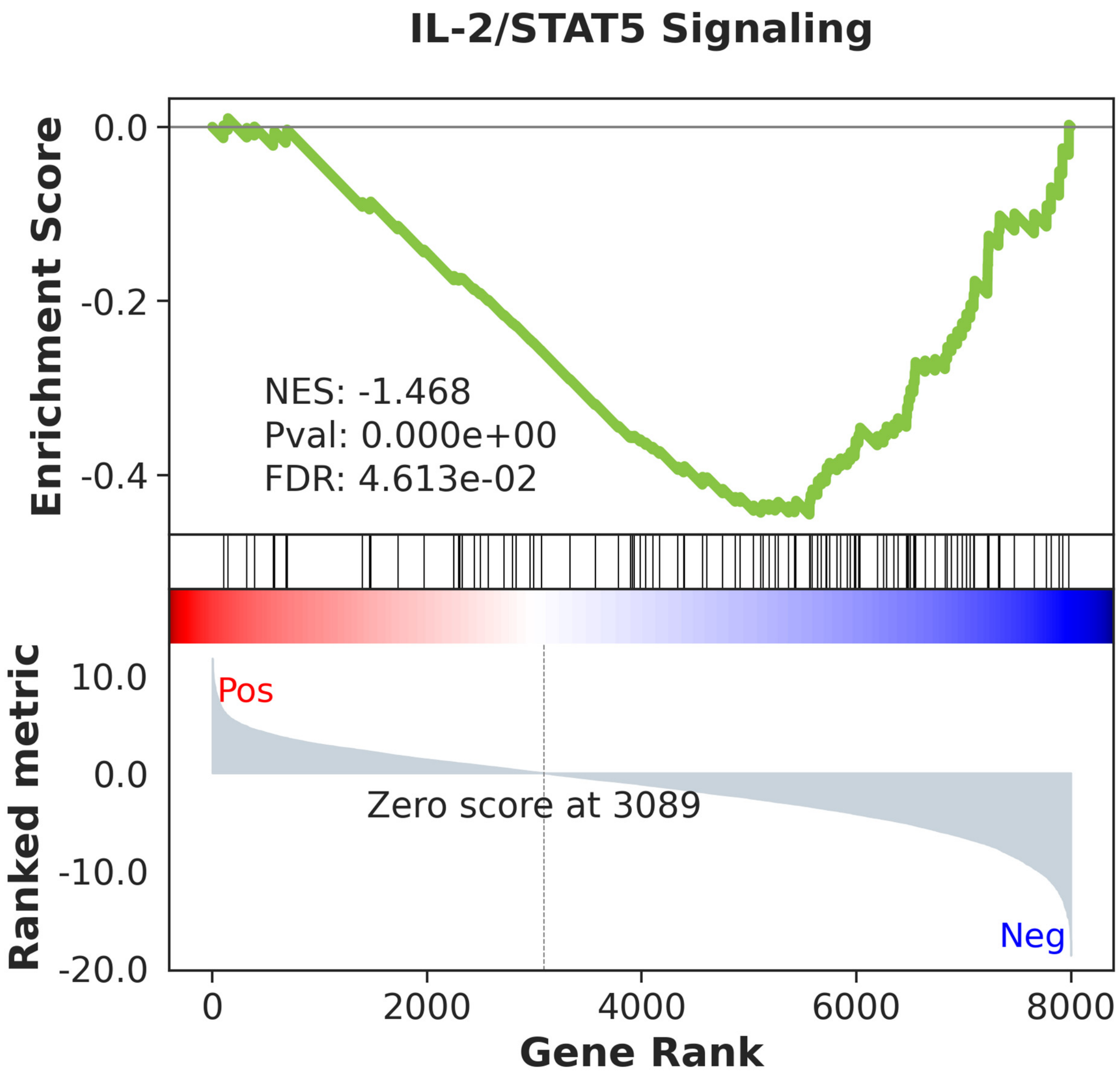

Figure S9. Individual GSEA enrichment plot for the IL-2/STAT5 Signaling Hallmark gene set. The plot shows three panels: (1) the running enrichment score (RES) curve, which peaks or troughs at the maximum enrichment score (ES); (2) vertical bars marking the positions of gene set members in the ranked gene list; and (3) a heat map of the ranked gene metric (Welch's t-statistic) along the list. The IL-2/STAT5 Signaling pathway was significantly enriched in Cluster 1 (Angiogenic/Inflammatory) (NES=−1.468, NOM p-value=<0.001, FDR q-value=0.046). Negative NES indicates that the leading-edge genes driving this enrichment are concentrated at the bottom of the ranked list, corresponding to genes with higher expression in Cluster 1.

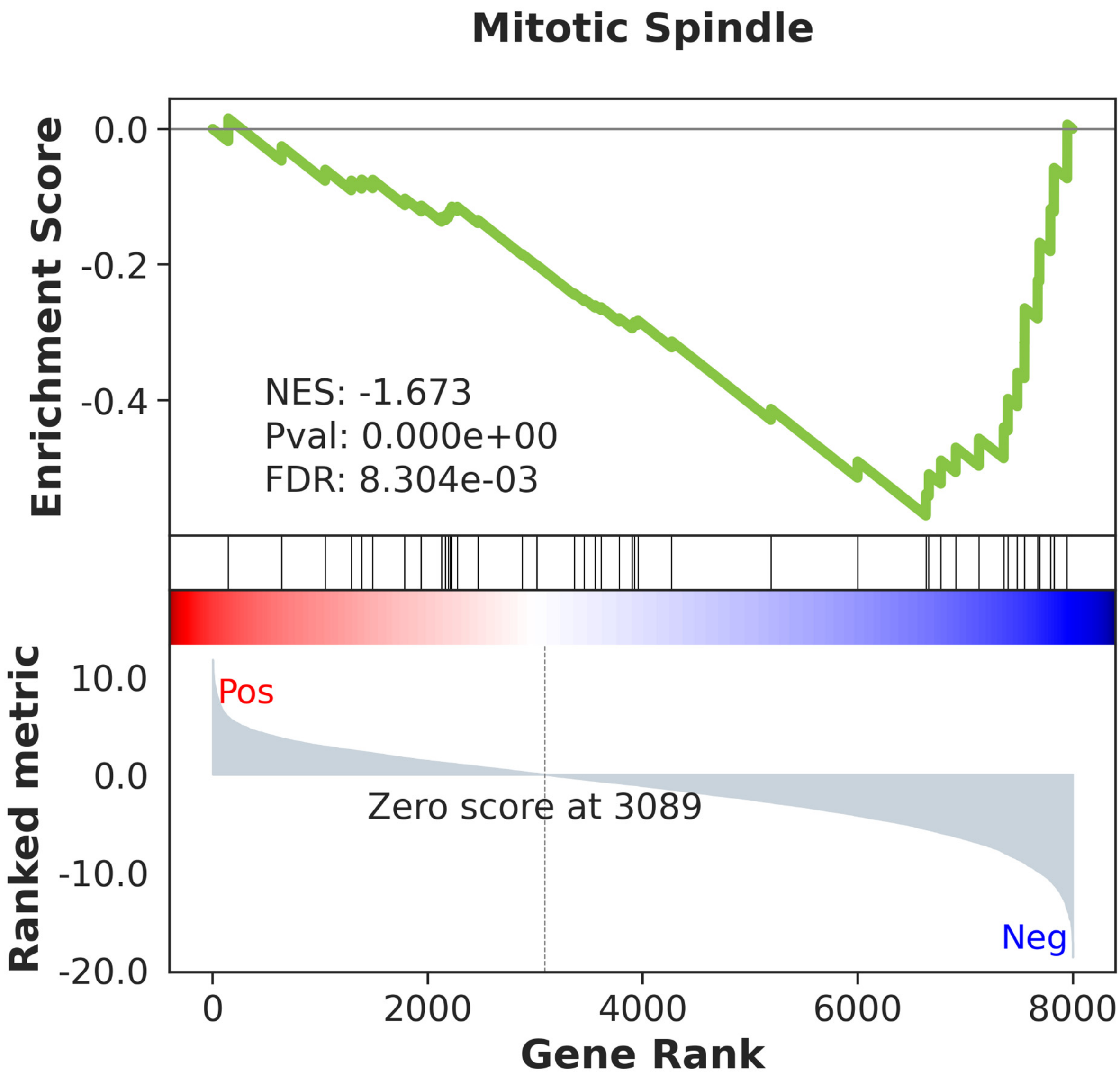

Figure S10. Individual GSEA enrichment plot for the Mitotic Spindle Hallmark gene set. The plot shows three panels: (1) the running enrichment score (RES) curve, which peaks or troughs at the maximum enrichment score (ES); (2) vertical bars marking the positions of gene set members in the ranked gene list; and (3) a heat map of the ranked gene metric (Welch's t-statistic) along the list. The Mitotic Spindle pathway was significantly enriched in Cluster 1 (Angiogenic/Inflammatory) (NES=−1.673, NOM p-value=<0.001, FDR q-value=0.008). Negative NES indicates that the leading-edge genes driving this enrichment are concentrated at the bottom of the ranked list, corresponding to genes with higher expression in Cluster 1.

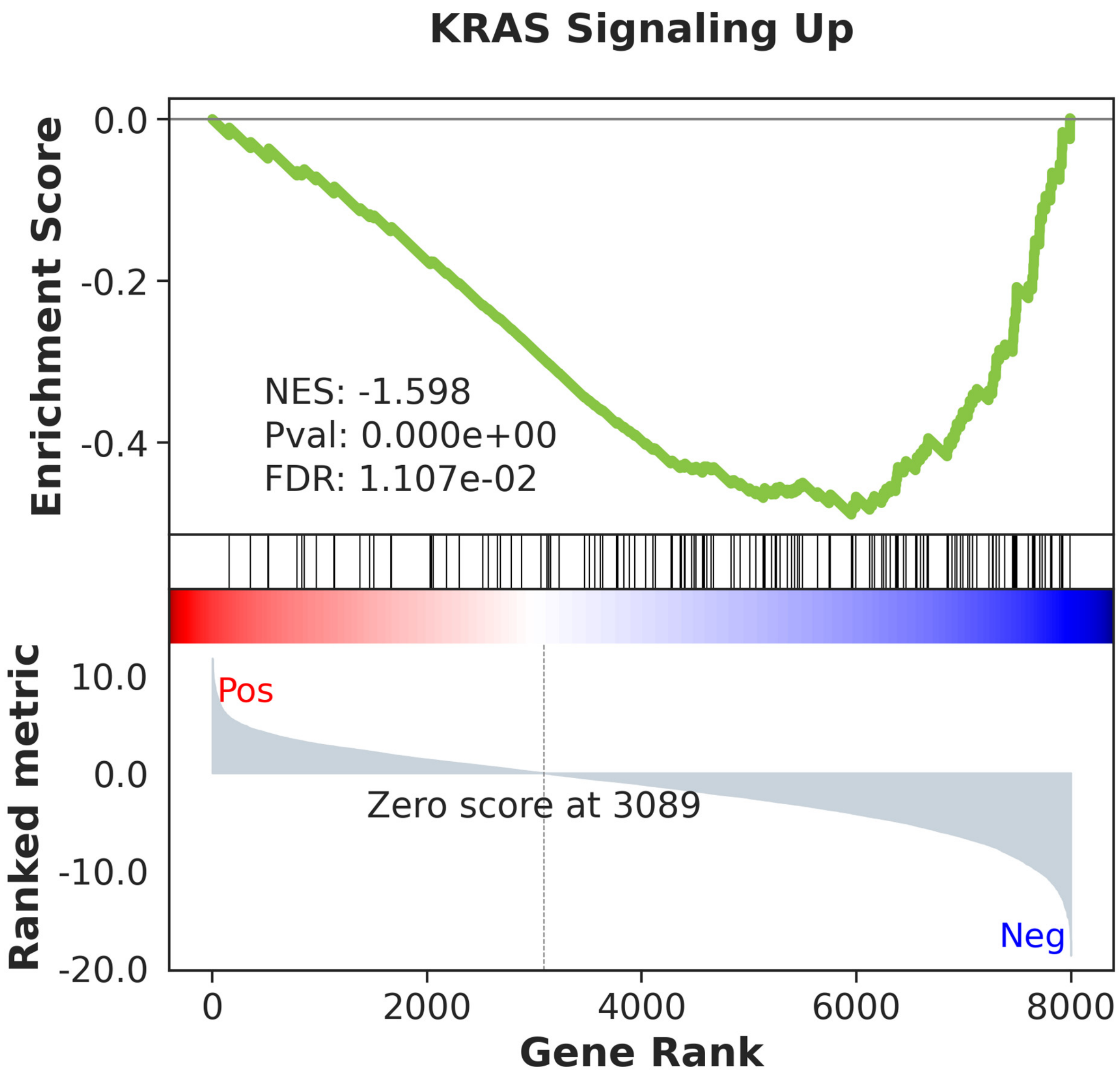

Figure S11. Individual GSEA enrichment plot for the KRAS Signaling Up Hallmark gene set. The plot shows three panels: (1) the running enrichment score (RES) curve, which peaks or troughs at the maximum enrichment score (ES); (2) vertical bars marking the positions of gene set members in the ranked gene list; and (3) a heat map of the ranked gene metric (Welch's t-statistic) along the list. The KRAS Signaling Up pathway was significantly enriched in Cluster 1 (Angiogenic/Inflammatory) (NES=−1.598, NOM p-value=<0.001, FDR q-value=0.011). Negative NES indicates that the leading-edge genes driving this enrichment are concentrated at the bottom of the ranked list, corresponding to genes with higher expression in Cluster 1.

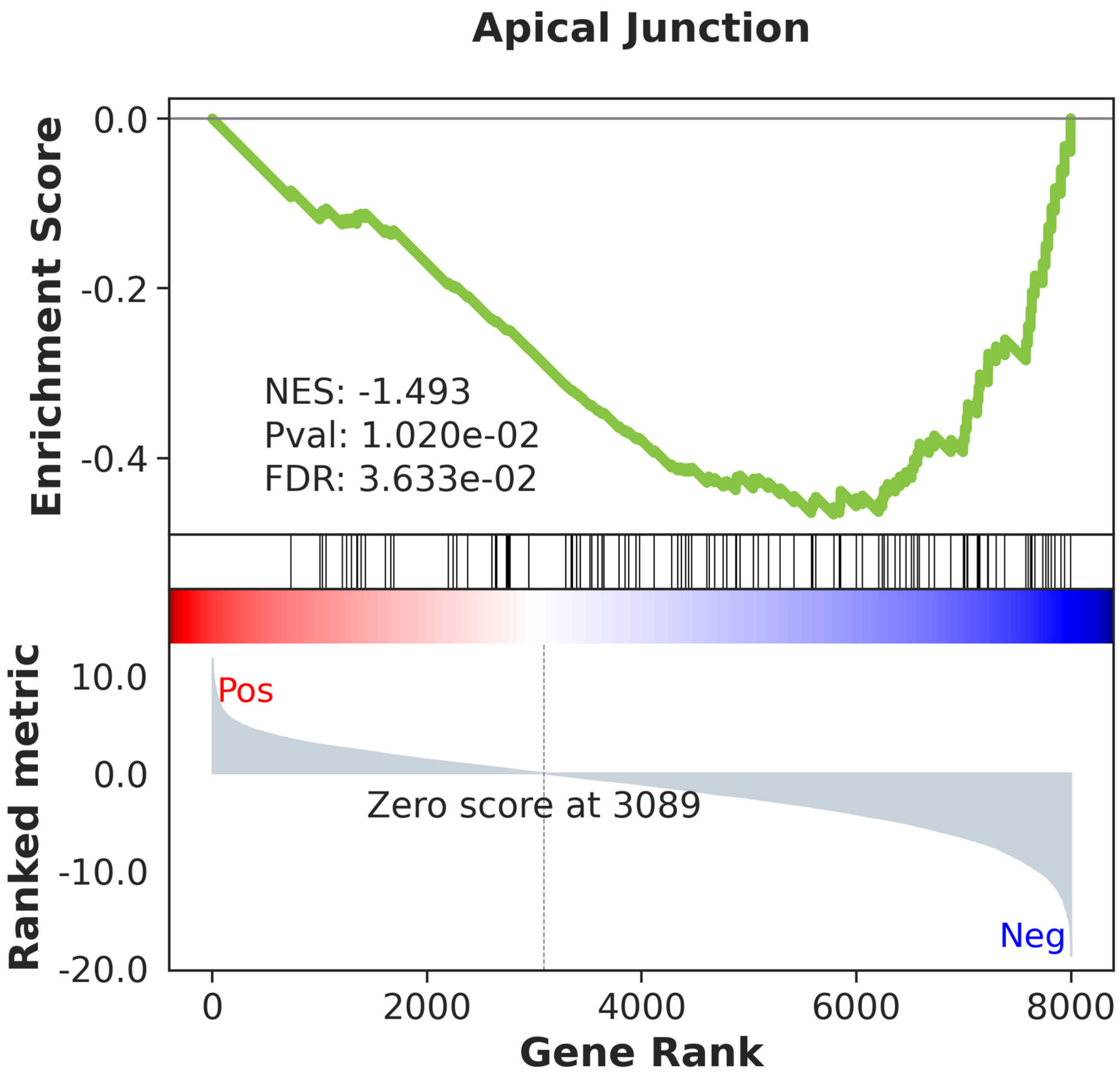

Figure S12. Individual GSEA enrichment plot for the Apical Junction Hallmark gene set. The plot shows three panels: (1) the running enrichment score (RES) curve, which peaks or troughs at the maximum enrichment score (ES); (2) vertical bars marking the positions of gene set members in the ranked gene list; and (3) a heat map of the ranked gene metric (Welch's t-statistic) along the list. The Apical Junction pathway was significantly enriched in Cluster 1 (Angiogenic/Inflammatory) (NES=−1.493, NOM p-value=0.010, FDR q-value=0.036). Negative NES indicates that the leading-edge genes driving this enrichment are concentrated at the bottom of the ranked list, corresponding to genes with higher expression in Cluster 1.

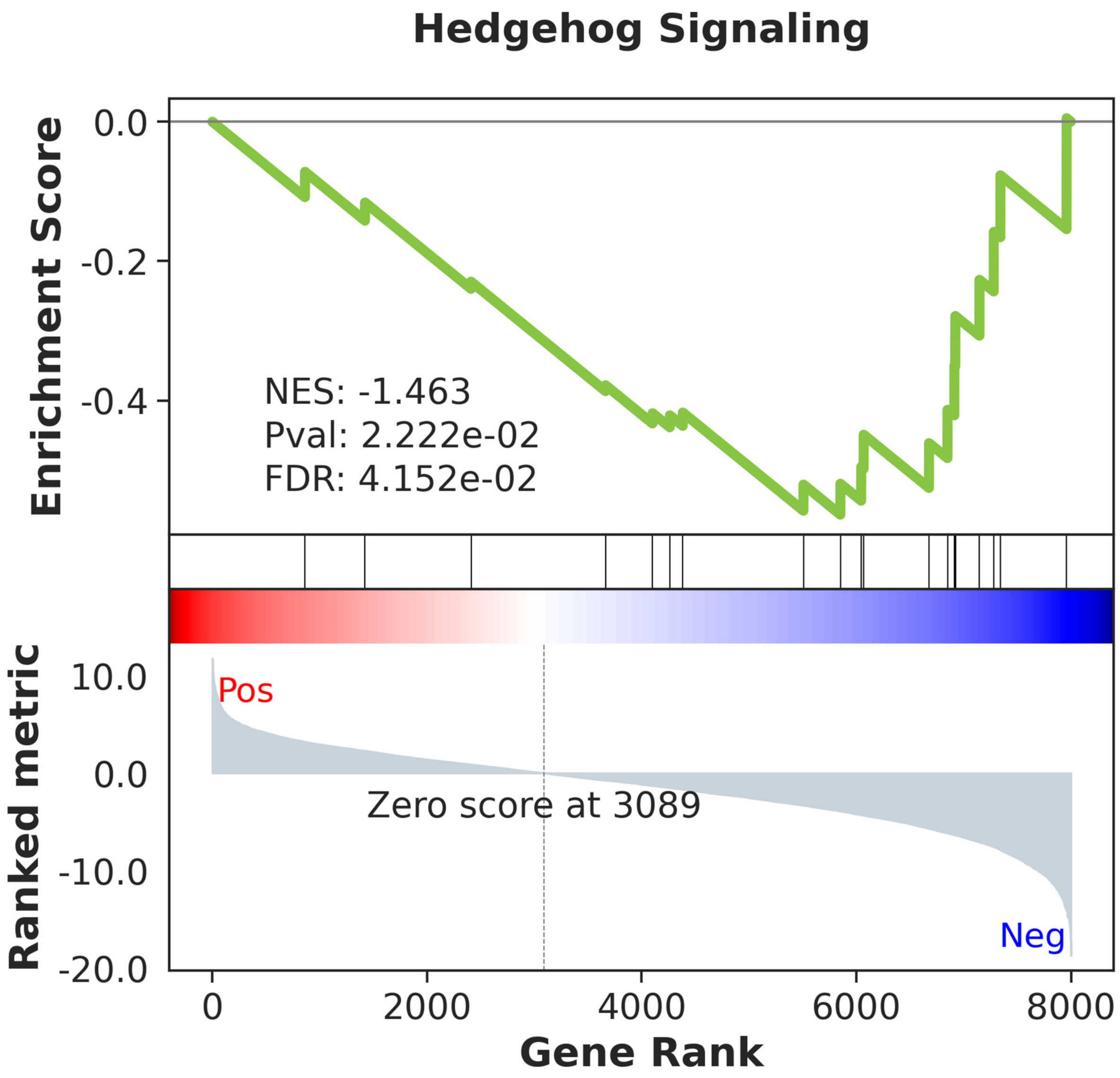

Figure S13. Individual GSEA enrichment plot for the Hedgehog Signaling Hallmark gene set. The plot shows three panels: (1) the running enrichment score (RES) curve, which peaks or troughs at the maximum enrichment score (ES); (2) vertical bars marking the positions of gene set members in the ranked gene list; and (3) a heat map of the ranked gene metric (Welch's t-statistic) along the list. The Hedgehog Signaling pathway was significantly enriched in Cluster 1 (Angiogenic/Inflammatory) (NES=−1.463, NOM p-value=0.022, FDR q-value=0.042). Negative NES indicates that the leading-edge genes driving this enrichment are concentrated at the bottom of the ranked list, corresponding to genes with higher expression in Cluster 1.

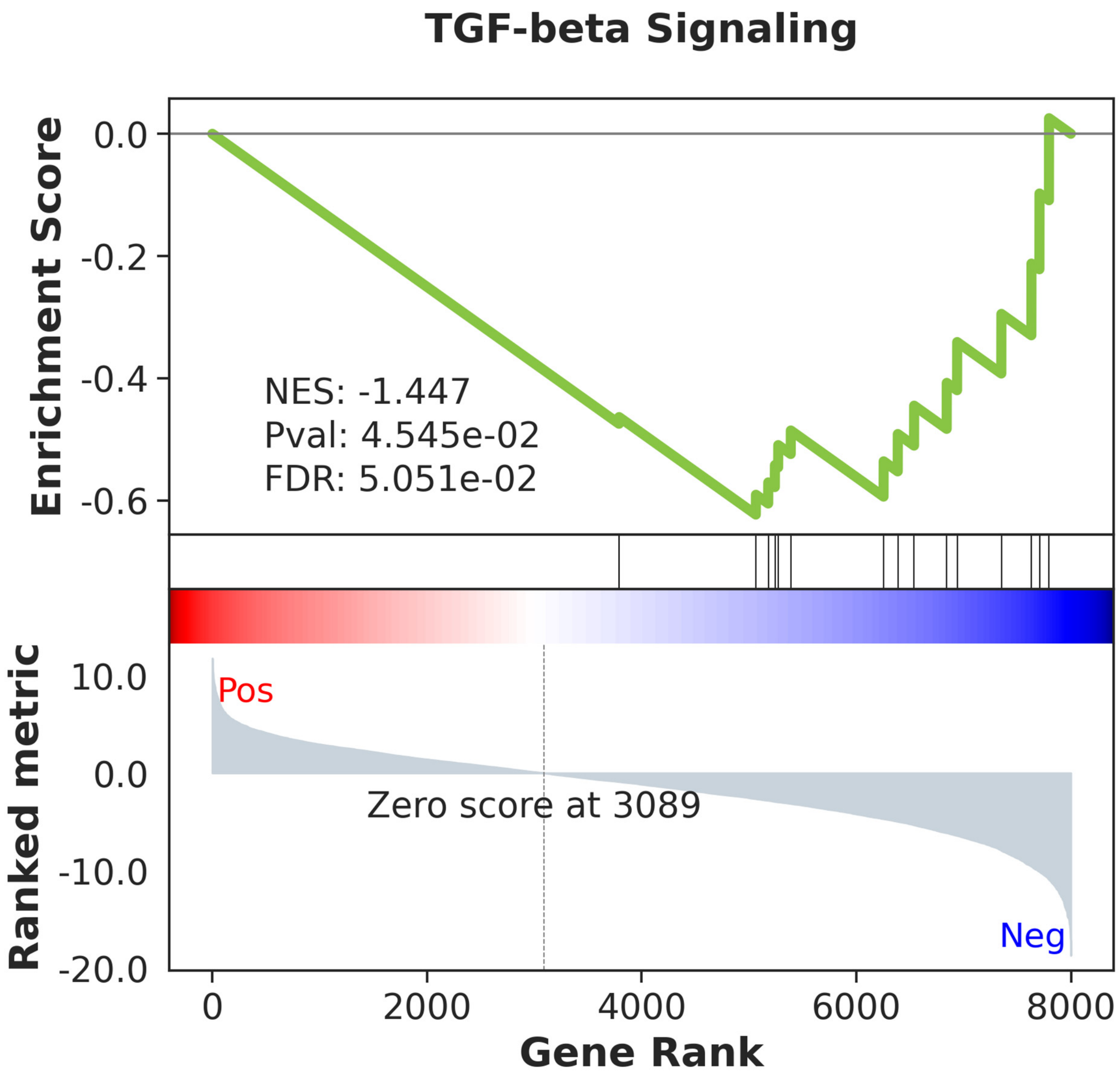

Figure S14. Individual GSEA enrichment plot for the TGF-beta Signaling Hallmark gene set. The plot shows three panels: (1) the running enrichment score (RES) curve, which peaks or troughs at the maximum enrichment score (ES); (2) vertical bars marking the positions of gene set members in the ranked gene list; and (3) a heat map of the ranked gene metric (Welch's t-statistic) along the list. The TGF-beta Signaling pathway was significantly enriched in Cluster 1 (Angiogenic/Inflammatory) (NES=−1.447, NOM p-value=0.045, FDR q-value=0.051). Negative NES indicates that the leading-edge genes driving this enrichment are concentrated at the bottom of the ranked list, corresponding to genes with higher expression in Cluster 1.

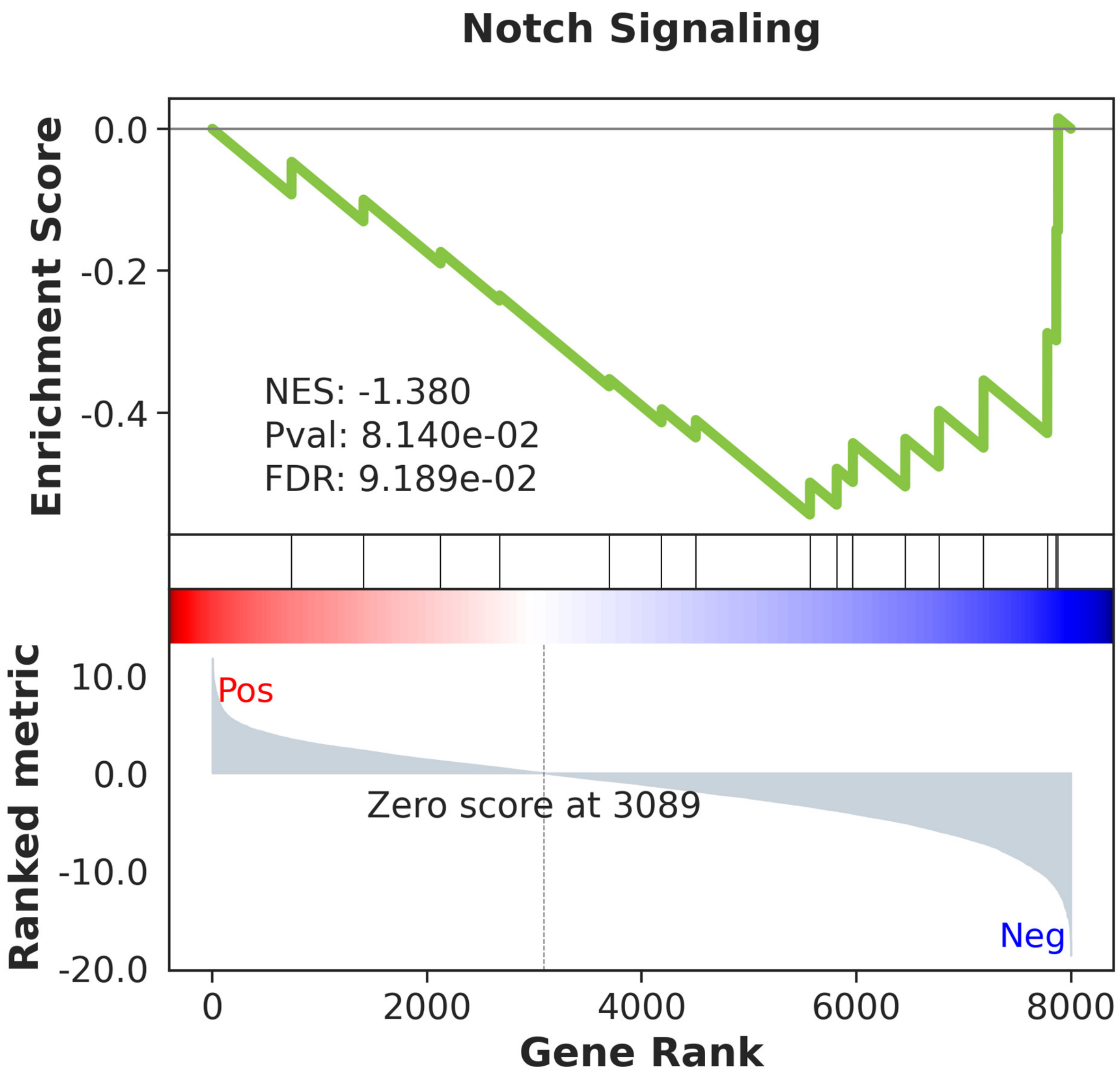

Figure S15. Individual GSEA enrichment plot for the Notch Signaling Hallmark gene set. The plot shows three panels: (1) the running enrichment score (RES) curve, which peaks or troughs at the maximum enrichment score (ES); (2) vertical bars marking the positions of gene set members in the ranked gene list; and (3) a heat map of the ranked gene metric (Welch's t-statistic) along the list. The Notch Signaling pathway was significantly enriched in Cluster 1 (Angiogenic/Inflammatory) (NES=−1.380, NOM p-value=0.081, FDR q-value=0.092). Negative NES indicates that the leading-edge genes driving this enrichment are concentrated at the bottom of the ranked list, corresponding to genes with higher expression in Cluster 1.

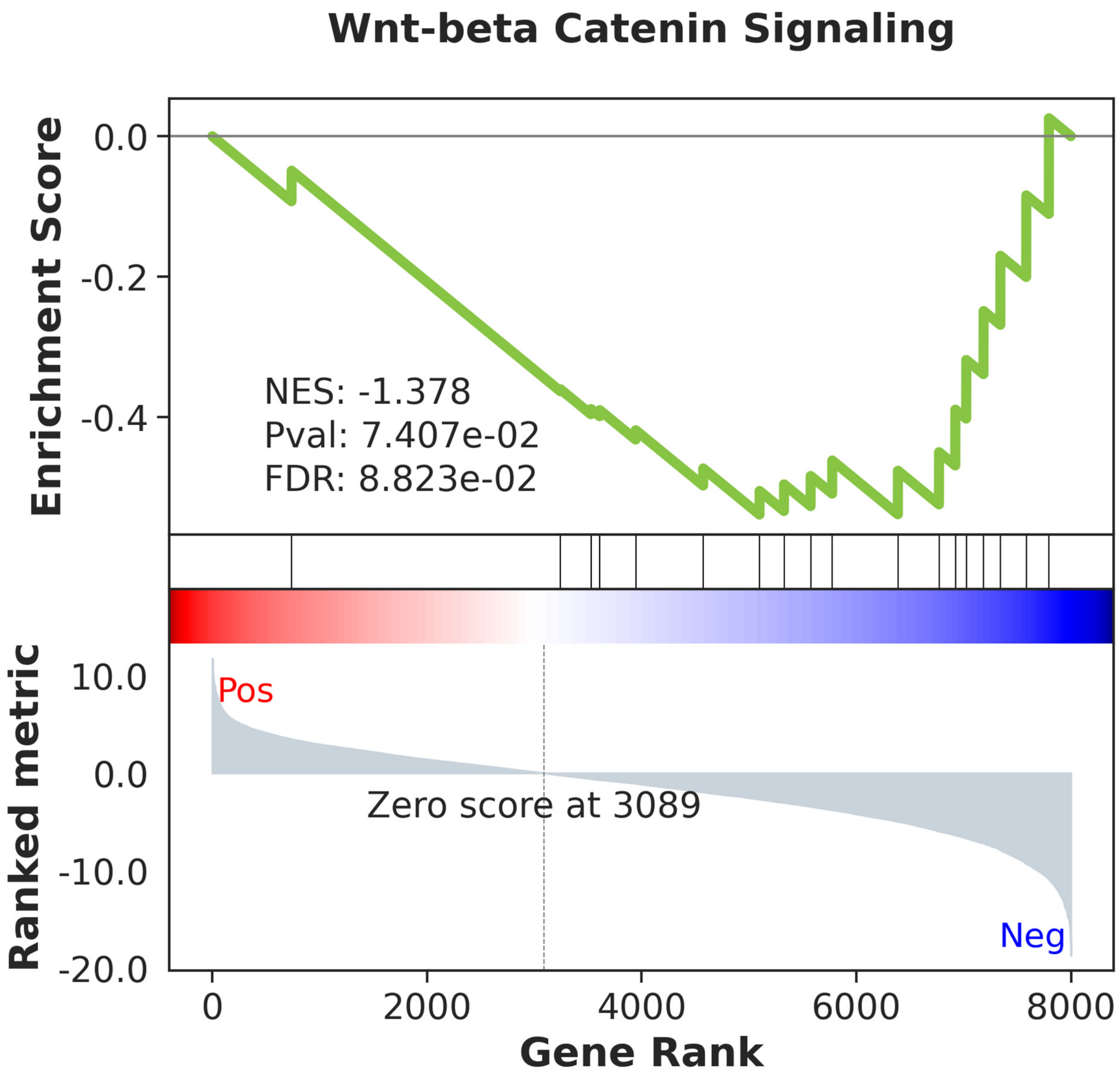

Figure S16. Individual GSEA enrichment plot for the Wnt-beta Catenin Signaling Hallmark gene set. The plot shows three panels: (1) the running enrichment score (RES) curve, which peaks or troughs at the maximum enrichment score (ES); (2) vertical bars marking the positions of gene set members in the ranked gene list; and (3) a heat map of the ranked gene metric (Welch's t-statistic) along the list. The Wnt-beta Catenin Signaling pathway was significantly enriched in Cluster 1 (Angiogenic/Inflammatory) (NES=−1.378, NOM p-value=0.074, FDR q-value=0.088). Negative NES indicates that the leading-edge genes driving this enrichment are concentrated at the bottom of the ranked list, corresponding to genes with higher expression in Cluster 1.

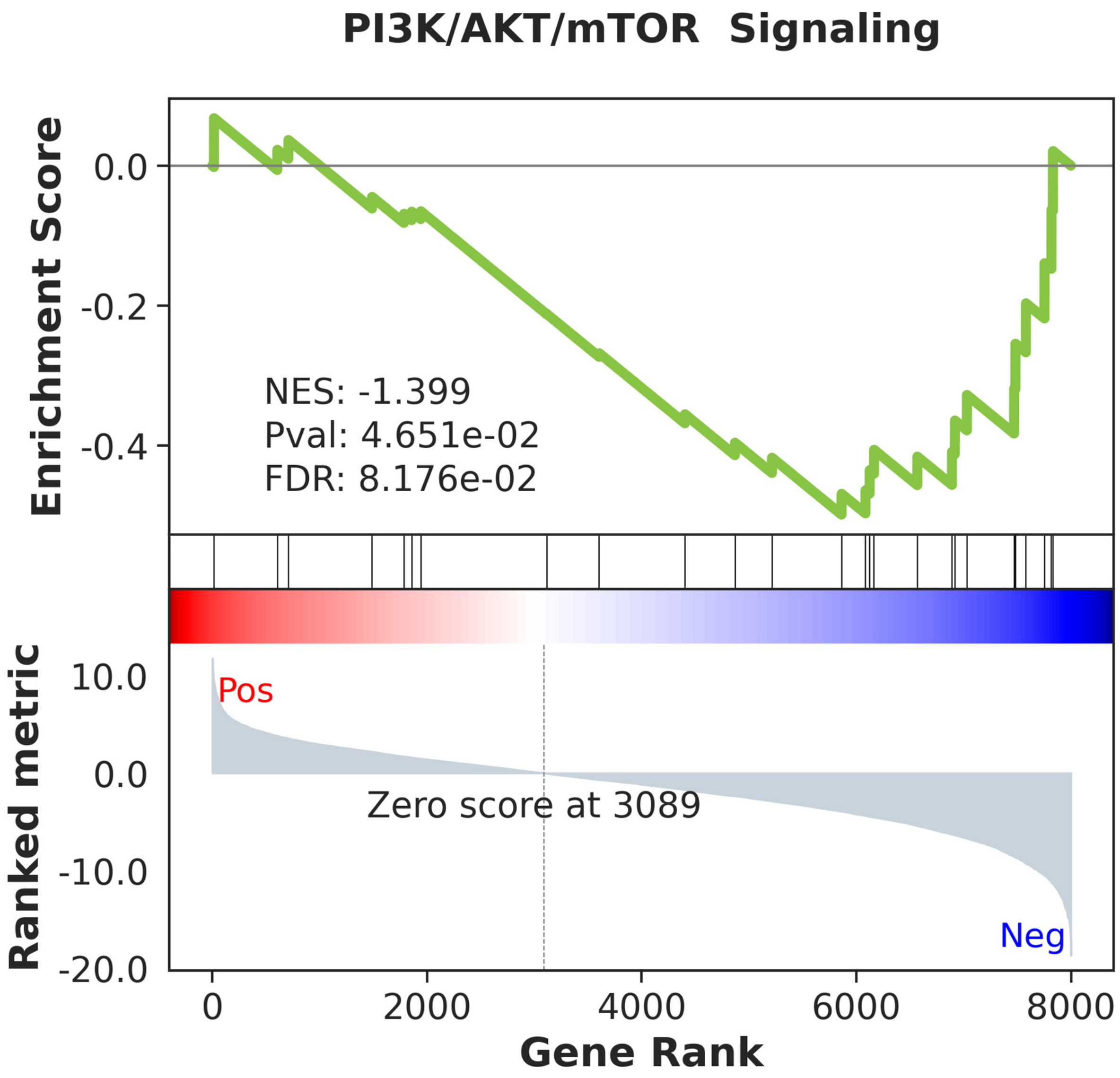

Figure S17. Individual GSEA enrichment plot for the PI3K/AKT/mTOR Signaling Hallmark gene set. The plot shows three panels: (1) the running enrichment score (RES) curve, which peaks or troughs at the maximum enrichment score (ES); (2) vertical bars marking the positions of gene set members in the ranked gene list; and (3) a heat map of the ranked gene metric (Welch's t-statistic) along the list. The PI3K/AKT/mTOR Signaling pathway was significantly enriched in Cluster 1 (Angiogenic/Inflammatory) (NES=−1.399, NOM p-value=0.047, FDR q-value=0.082). Negative NES indicates that the leading-edge genes driving this enrichment are concentrated at the bottom of the ranked list, corresponding to genes with higher expression in Cluster 1.

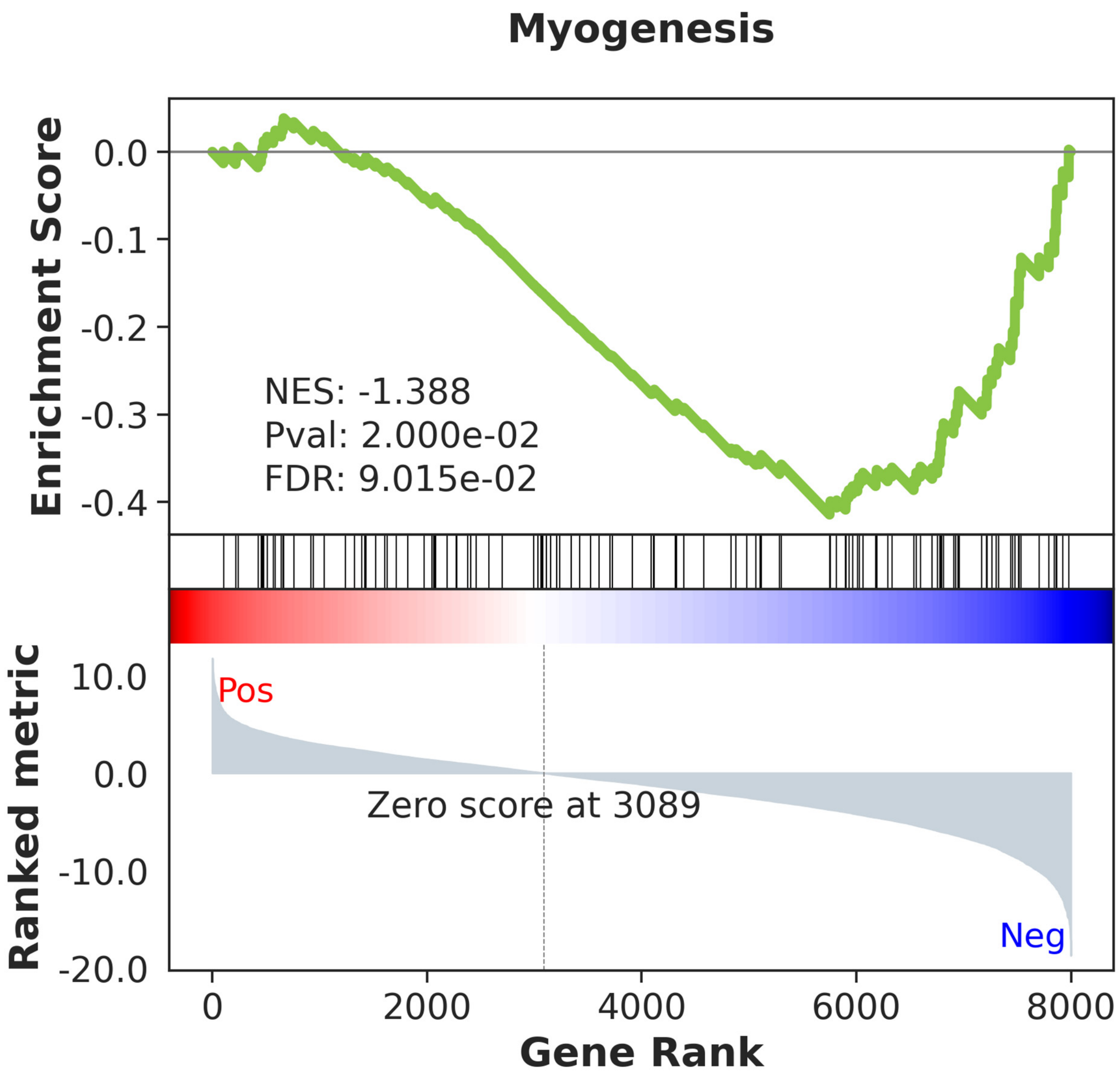

Figure S18. Individual GSEA enrichment plot for the Myogenesis Hallmark gene set. The plot shows three panels: (1) the running enrichment score (RES) curve, which peaks or troughs at the maximum enrichment score (ES); (2) vertical bars marking the positions of gene set members in the ranked gene list; and (3) a heat map of the ranked gene metric (Welch's t-statistic) along the list. The Myogenesis pathway was significantly enriched in Cluster 1 (Angiogenic/Inflammatory) (NES=−1.388, NOM p-value=0.020, FDR q-value=0.090). Negative NES indicates that the leading-edge genes driving this enrichment are concentrated at the bottom of the ranked list, corresponding to genes with higher expression in Cluster 1.

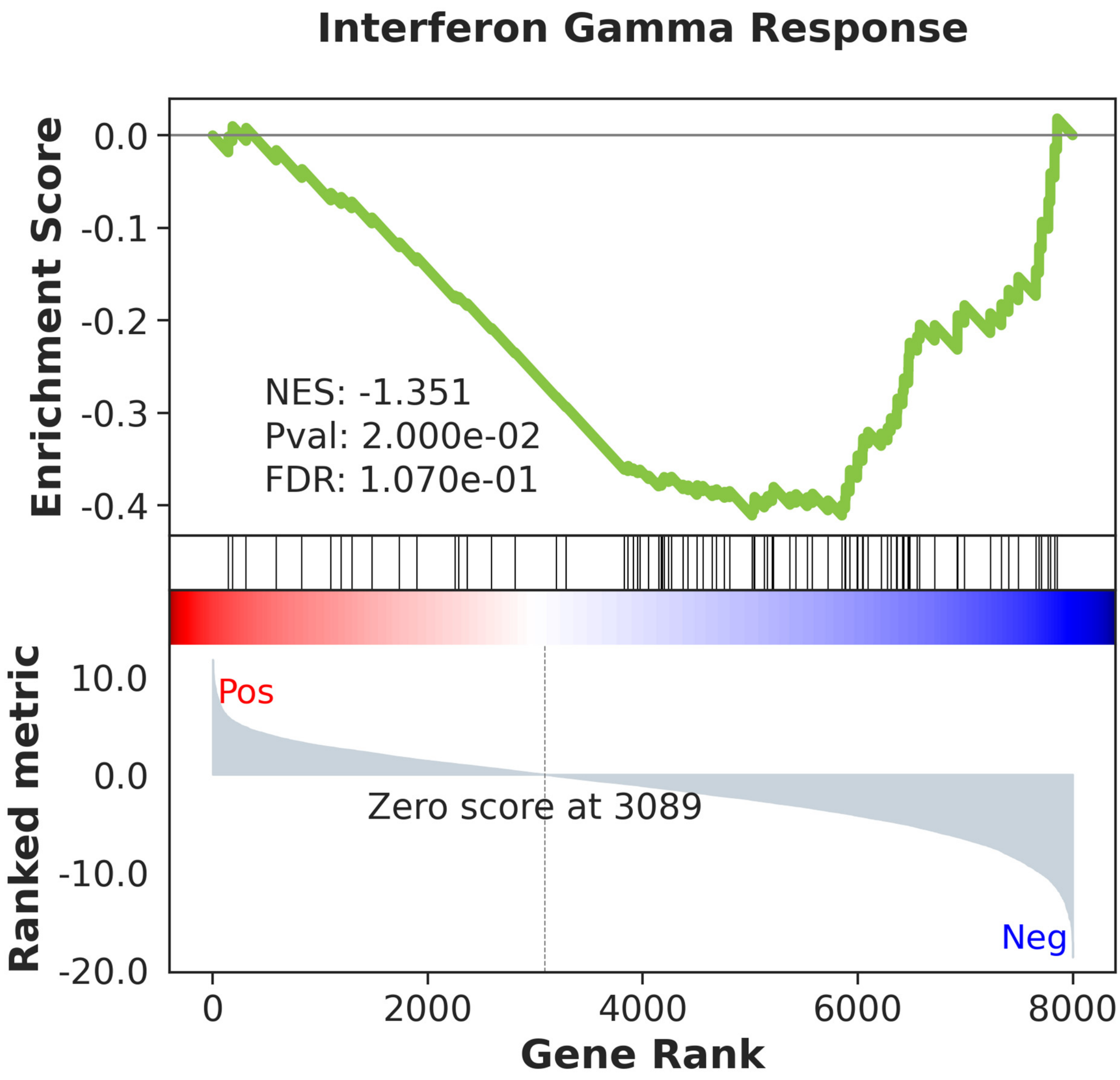

Figure S19. Individual GSEA enrichment plot for the Interferon Gamma Response Hallmark gene set. The plot shows three panels: (1) the running enrichment score (RES) curve, which peaks or troughs at the maximum enrichment score (ES); (2) vertical bars marking the positions of gene set members in the ranked gene list; and (3) a heat map of the ranked gene metric (Welch's t-statistic) along the list. The Interferon Gamma Response pathway was significantly enriched in Cluster 1 (Angiogenic/Inflammatory) (NES=−1.351, NOM p-value=0.020, FDR q-value=0.107). Negative NES indicates that the leading-edge genes driving this enrichment are concentrated at the bottom of the ranked list, corresponding to genes with higher expression in Cluster 1.

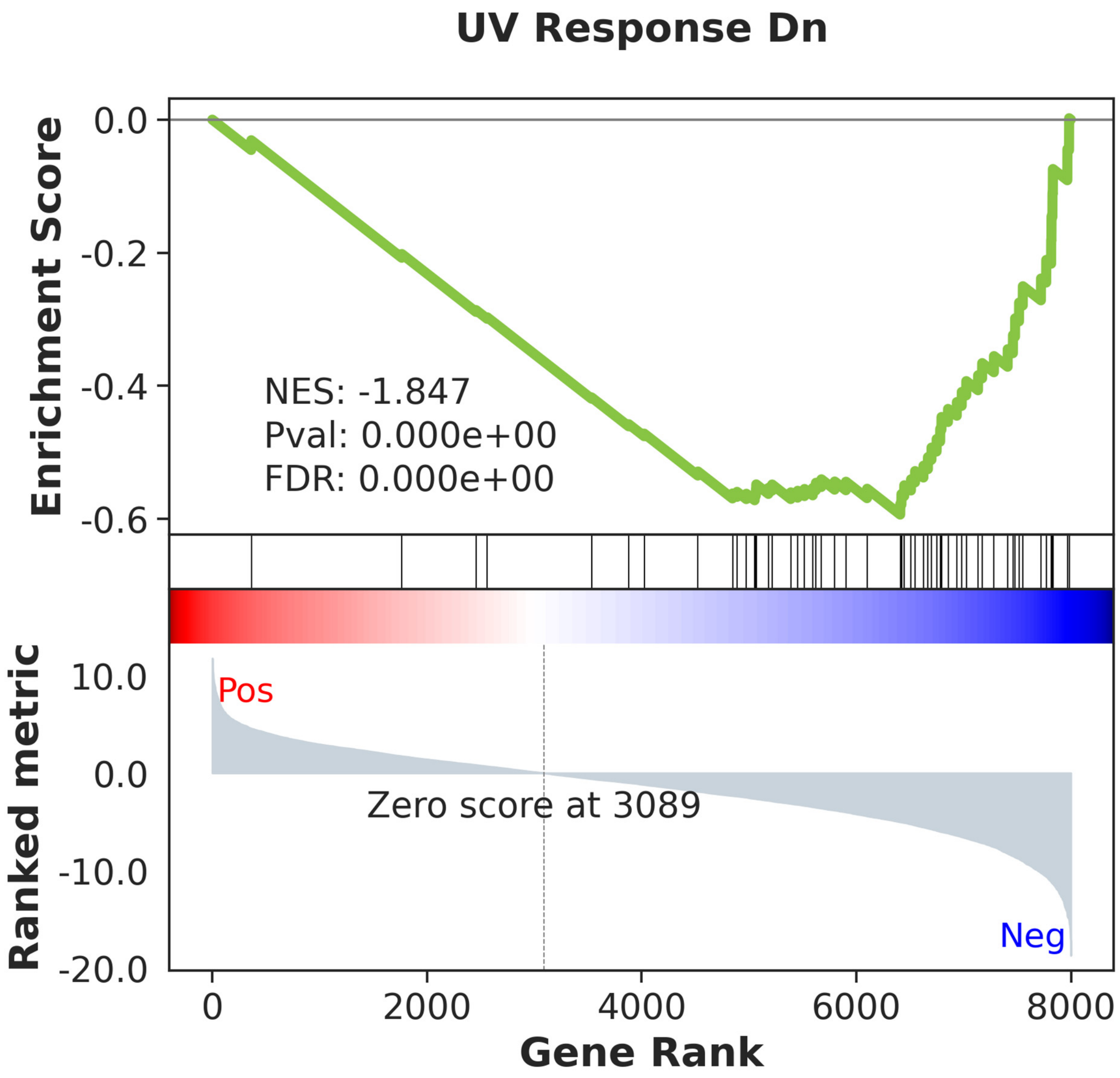

Figure S20. Individual GSEA enrichment plot for the UV Response Dn Hallmark gene set. The plot shows three panels: (1) the running enrichment score (RES) curve, which peaks or troughs at the maximum enrichment score (ES); (2) vertical bars marking the positions of gene set members in the ranked gene list; and (3) a heat map of the ranked gene metric (Welch's t-statistic) along the list. The UV Response Dn pathway was significantly enriched in Cluster 1 (Angiogenic/Inflammatory) (NES=−1.847, NOM p-value=<0.001, FDR q-value=<0.001). Negative NES indicates that the leading-edge genes driving this enrichment are concentrated at the bottom of the ranked list, corresponding to genes with higher expression in Cluster 1.

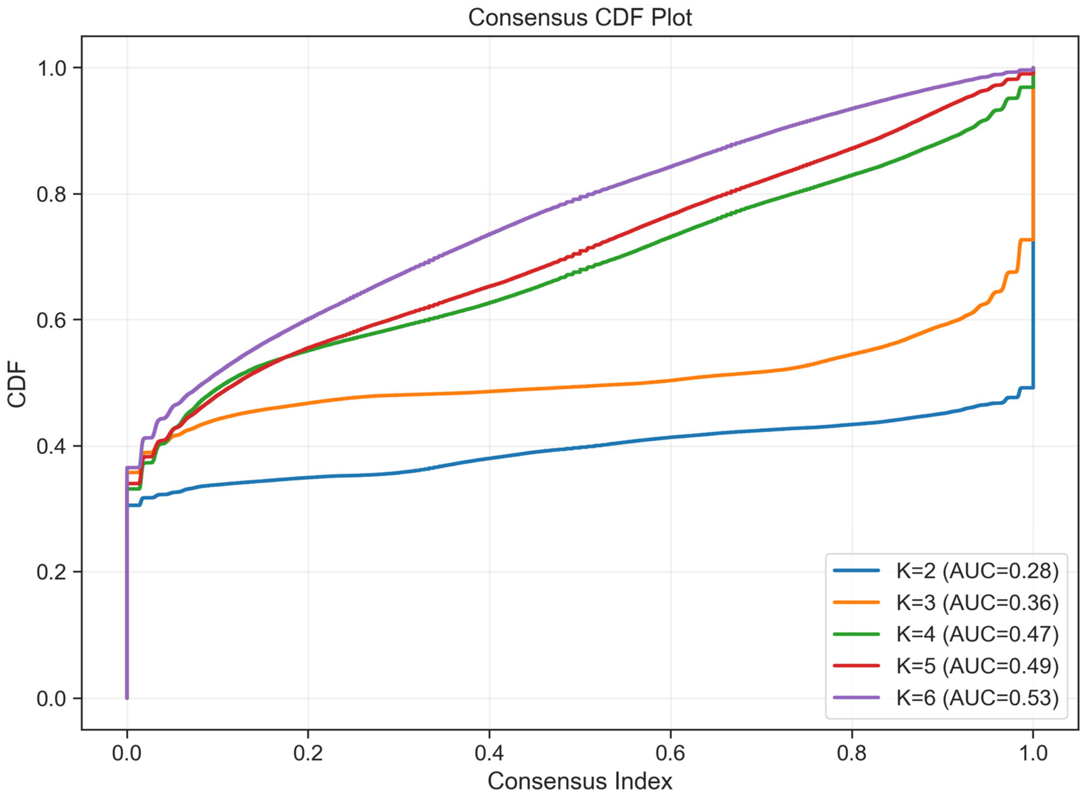

Figure S21. Consensus Cumulative Distribution Function (CDF) plots for K=2 to K=6
